# Comparative Evaluation of Open-Source Bioinformatics Pipelines for Full-Length Viral Genome Assembly

**DOI:** 10.3390/v16121824

**Published:** 2024-11-24

**Authors:** Levente Zsichla, Marius Zeeb, Dávid Fazekas, Éva Áy, Dalma Müller, Karin J. Metzner, Roger D. Kouyos, Viktor Müller

**Affiliations:** 1 Institute of Biology, ELTE Eötvös Loránd University, 1117 Budapest, Hungary; zsichla.levente@ttk.elte.hu (L.Z.); fazekasda@gmail.com (D.F.); dalma.muller2@gmail.com (D.M.); 2 National Laboratory for Health Security, ELTE Eötvös Loránd University, 1117 Budapest, Hungary; ay.eva@nngyk.gov.hu; 3 Department of Infectious Diseases and Hospital Epidemiology, University Hospital of Zurich, University of Zurich, 8091 Zurich, Switzerland; marius.zeeb@usz.ch (M.Z.); karin.metzner@usz.ch (K.J.M.); roger.kouyos@usz.ch (R.D.K.); 4 Institute of Medical Virology, University of Zurich, 8057 Zurich, Switzerland; 5 Earlham Institute, Norwich NR4 7UZ, UK; 6 National Reference Laboratory for Retroviruses, Department of Virology, National Center for Public Health and Pharmacy, 1097 Budapest, Hungary; 7 Department of Bioinformatics, Semmelweis University, 1094 Budapest, Hungary

**Keywords:** next-generation sequencing, genome assembly, bioinformatics pipeline, benchmarking

## Abstract

The increasingly widespread application of next-generation sequencing (NGS) in clinical diagnostics and epidemiological research has generated a demand for robust, fast, automated, and user-friendly bioinformatics workflows. To guide the choice of tools for the assembly of full-length viral genomes from NGS datasets, we assessed the performance and applicability of four open-source bioinformatics pipelines (shiver—for which we created a user-friendly Dockerized version, referred to as dshiver; SmaltAlign; viral-ngs; and V-pipe) using both simulated and real-world HIV-1 paired-end short-read datasets and default settings. All four pipelines produced consensus genome assemblies with high quality metrics (genome fraction recovery, mismatch and indel rates, variant calling F1 scores) when the reference sequence used for assembly had high similarity to the analyzed sample. The shiver and SmaltAlign pipelines (but not viral-ngs and V-Pipe) also showed robust performance with more divergent samples (non-matching subtypes). With empirical datasets, SmaltAlign and viral-ngs exhibited an order of magnitude shorter runtime compared to V-Pipe and shiver. In terms of applicability, V-Pipe provides the broadest functionalities, SmaltAlign and dshiver combine user-friendliness with robustness, while the use of viral-ngs requires less computational resources compared to other pipelines. In conclusion, if a closely matched reference sequence is available, all pipelines can reliably reconstruct viral consensus genomes; therefore, differences in user-friendliness and runtime may guide the choice of the pipeline in a particular setting. If a matched reference sequence cannot be selected, we recommend shiver or SmaltAlign for robust performance. The new Dockerized version of shiver offers ease of use in addition to the accuracy and robustness of the original pipeline.

## 1. Introduction

The clinical diagnostics and molecular epidemiology of viral infections rely increasingly on next-generation sequencing (NGS) due to its speed, high-throughput performance, and cost-effectiveness. The Advanced Molecular Detection program in the US applies NGS technologies in nearly every area of infectious disease public health [1]. A largely NGS-based surveillance of the COVID-19 (coronavirus disease 2019) pandemic yielded more than 8 million full-length severe acute respiratory syndrome coronavirus 2 (SARS-CoV-2) genomic sequences [2], and human immunodeficiency virus 1 (HIV-1) genotypic resistance testing is also transitioning to NGS technologies in clinical diagnostics [3,4,5]. NGS has also simplified the sequencing of complete viral genomes, allowing for the detection of mutations outside the traditional target regions for drug resistance genotyping [6,7,8,9,10,11,12], and for co-receptor tropism prediction [13,14,15,16,17]. The use of full-length genomes also facilitates the identification of transmission clusters [18,19], subtyping [20], and the detection of recombinant forms [21,22].

Notably, consensus genomes assembled from NGS data can be used as input data for all tools developed for Sanger sequences [23], while minority variant calling (from reads mapped to the consensus sequence) enhances drug resistance prediction [24,25,26,27] and enables more precise assessments of infection recency [28] or transmission patterns [29,30,31].

Genome assemblers typically rely on two traditional approaches: reference-based and de novo genome assembly. Reference-based assembly involves mapping sequencing reads to an existing reference sequence and identifying the majority base at each genomic position. This approach is faster, computationally more efficient, and robustly generates full-length genomic sequences by imputation where coverage is low. However, this method is sensitive to the choice of reference and may introduce a bias into read mapping by discarding reads that are divergent from the reference sequence, potentially missing novel information in the sample. In contrast, by constructing the consensus sequence autonomously from the overlapping reads, de novo assembly avoids such bias, but it is unable to bridge gaps if non-overlapping contigs are produced.

The quality of de novo genome assemblies is traditionally evaluated in three key dimensions, often referred to as the three Cs [32]. The first is contiguity, which considers the number and lengths of the recovered genome fragments; however, it is important to note that these metrics (such as N50, NG50, or the number and length of contigs) are not relevant for assemblies generated through reference-based or hybrid approaches, resulting in a single contiguous sequence. Completeness, the second aspect, measures the proportion of the genome successfully reconstructed during assembly, typically encompassing all nucleotide sites (genome fraction recovery) or conserved genes (e.g., Benchmarking Universal Single-Copy Orthologs scores [33]). Lastly, correctness evaluates the assembly’s precision in terms of mismatches, indels, or misassemblies when compared to a gold-standard reference assembly. 

Recently, several solutions have been developed to combine the advantages of de novo and reference-based assembly [34,35,36,37], but these hybrid genome assembly pipelines have not been comprehensively evaluated. We assessed the performance of four open-source bioinformatics pipelines (shiver, SmaltAlign, viral-ngs, and V-pipe) used for assembling full-length viral genomes from Illumina short reads. In addition to measures of genome quality and its effect on downstream variant calling analysis, we also evaluated technical benchmarks that affect practical utility (runtime and memory usage). To ensure a comprehensive evaluation, we utilized both simulated and empirical HIV-1 datasets. With this study, we aim to help the selection of a viral genome assembly pipeline from free-to-use open-source solutions both in research and potentially in clinical settings.

## 2. Materials and Methods

### 2.1. Datasets

#### 2.1.1. Simulated HIV-1 Quasispecies Composition (SIM)

To generate an in silico population of full-length HIV-1 genomic sequences, we utilized SANTA-SIM [38] with the initial user-supplied sequences described below. Our customized HIV-1 configuration implemented point mutations (mean 2.5 × 10^−5^/base/generation [39] multiplied by relative in vivo mutation rates for all 12 nucleotide combinations [40]), indels (3 × 10^−5^/base/generation [41]), and recombination events (0.1 dual infection and 1 × 10^−5^ recombination per generation [42]), with a constant effective population size of 1000 [43]. The simulations spanned a randomly selected duration between 50 and 1500 generations to generate samples exhibiting diversity patterns resembling both recent and chronic infections.

To cover the genetic variability of HIV-1, we selected four distinct HIV-1 group M subtype consensus sequences (A1, B, C, and CRF01_AE) and one group O consensus sequence (from the HIV-1 consensus sequence alignment of the LANL HIV database [44,45]) for in silico quasispecies simulation. This selection also enabled us to examine the effect of an increased divergence of the analyzed sample from the reference genome on the performance of the pipelines (some of which use the HXB2, GenBank: K03455.1, isolate as a fixed reference sequence). Additionally, to show whether providing a well-matched reference sequence alleviates the effect, we included an additional scenario in which genome assemblers used the consensus group O sequence as a reference for the assembly of the in silico group O samples. To incorporate further crucial aspects of laboratory work, we also added two different read coverage scenarios (500 and 10,000 per base coverage) and the absence or presence of laboratory contamination to our simulations. The presence of laboratory contamination was simulated by introducing 8600 randomly generated read pairs, which represented approximately 5% and 100% of the number of reads in the high- and low-coverage scenarios, respectively. These read pairs were derived from a 55 kb fragment of human chromosome 19 (GRCh38.p14, chr22:58283717-58338638) including the 11 kb Endogenous Retrovirus Group K3 Member 1 (*ERVK-1*) gene. This procedure resulted in 24 unique parameter combinations (5 + 1 subtype/reference, 2 coverage, and 2 contamination scenarios) with 20 replicates each, totaling 480 simulations.

#### 2.1.2. Single-Genome Sequencing Data (SGS-FULL)

Single-genome amplification and sequencing (SGS) is used to characterize the within-host genetic diversity of chronic HIV infections [46]. The selected SGS dataset contained 13–55 intact, near-full-length (~8800 bp) subtype B genomic sequences from each of 5 patients on antiretroviral therapy, 4 of whom were undergoing treatment interruption during the study period [47]. 

#### 2.1.3. Sanger and Next-Generation Sequencing from the Same Sample (SS+NGS)

The results of Sanger sequencing, due to the low per base error rate of the instrument (0.0001%) [48], can serve as benchmarks for assessing genome assemblies generated from short-read NGS data. We used unpublished sequence data obtained from parallel Sanger and next-generation sequencing of the same samples to enable such comparisons.

Plasma samples were collected from ART-naive patients diagnosed as HIV-positive between 2016 and 2022 at the Center for HIV, Central Hospital of Southern Pest, National Institute of Hematology and Infectious Disease, Budapest, Hungary. HIV-1 RNA extraction; the amplification of protease, reverse transcriptase, and integrase regions; and details of the Sanger sequencing method were described previously [49,50,51]. Additionally, 41 samples were selected for next-generation sequencing using a protocol developed for the amplification of near-full-length HIV-1 genome and short-read sequencing based on previous publications [52] (see Supplementary Materials), resulting in 46 NGS datasets from 41 samples (Illumina MiSeq platform; paired-end 2 × 150 bp library configuration). The subtype distribution of sequences determined by REGA (v3.46) [53] from the consensus sequences produced by dshiver was as follows: B (22.0%), CRF 01_AE (9.76%), F1 (7.3%), CRF 19_cpx (4.9%), B-like (4.9%), A1 (2.4%), C-like (2.4%), and other recombinant forms (46.3%).

In sequence positions where Sanger sequencing produced ambiguous results or NGS data strongly indicated a different base call (allele frequency over 0.7), we corrected the sequence to prevent bias in the benchmarking results due to inconsistencies between the two datasets.

#### 2.1.4. Next-Generation Sequencing Dataset for Runtime Benchmarking (NGS-FULL)

We utilized a publicly available NGS dataset [52] to investigate how the computational demands of each genome assembler scale with increasing dataset size. The chosen dataset comprised NGS data from 92 plasma samples, each subjected to amplification in four overlapping segments covering nearly the entire HIV-1 genome (~8800 bp). Subsequently, the samples were sequenced using the Illumina MiSeq platform, generating 175,302 to 1,649,546 paired-end reads (2 × 250 bp) per sample.

### 2.2. Workflow

#### 2.2.1. Construction of Benchmarking References

**Figure 1:**
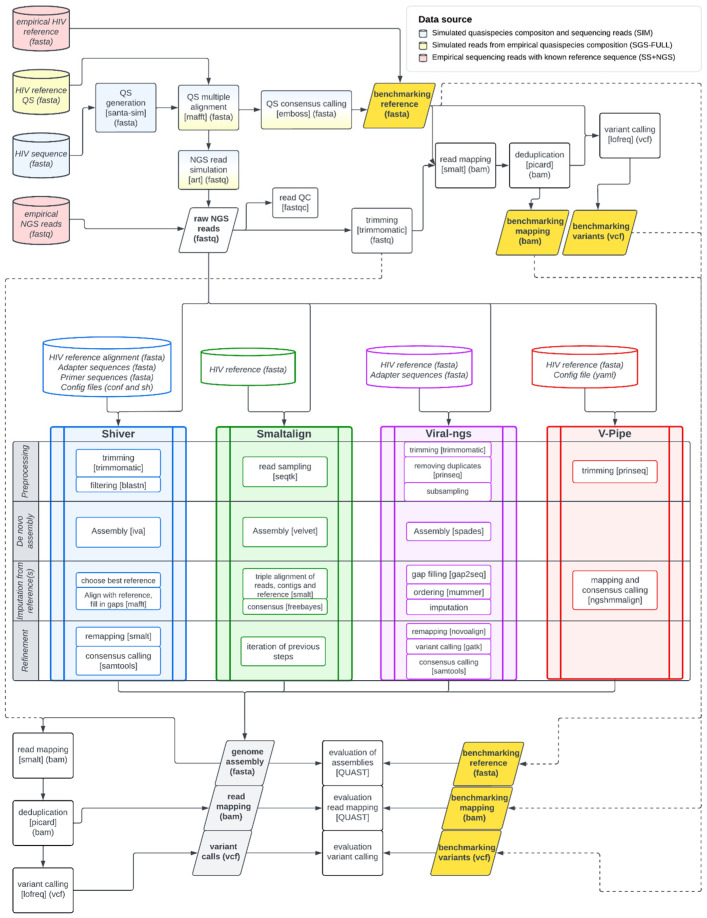
Detailed workflow of the benchmarking pipeline: Data sources are depicted as cylindrical boxes with red (SS+NGS dataset), yellow (SGS-FULL dataset), and blue (SIM dataset) coloring, depending on the specific dataset analyzed. Analysis steps are shown as rectangles, and important files as parallelograms. Within the rectangles, tools used in each analysis step are shown in square brackets, and file extensions are indicated in parentheses. For the SS+NGS dataset, the benchmarking reference sequence and raw read files were directly supplied. For the SGS-FULL dataset, the benchmarking reference was estimated using the consensus of all sequenced viral genomes, and the sequencing reads were simulated based on all viral variants. In the case of the SIM dataset, both data types were simulated. The raw reads were then preprocessed and mapped to the benchmarking sequence, and viral variants were identified to produce the benchmarking mapping and variant files. After that, the raw reads and configuration files were supplied for each pipeline to perform genome assembly. The preprocessed reads were mapped to these genome assemblies, and minority variants were identified for each tool separately. Finally, the consensus genomes and downstream analysis files were compared to the benchmarking datasets to obtain benchmarking quality metrics. All information on the pipeline, the developed in-house scripts, and their parametrization is available in the GitHub repository of this project at https://github.com/hcovlab/ViralNGSBenchmarking (accessed on 23 November 2024). Abbreviations: QS—quasispecies, QC—quality control.

For each simulated or empirical sample, we constructed the consensus sequence of all variants present in the original sample (in the case of the SIM dataset, the generated quasispecies) to use as a benchmarking reference (Figure 1). We performed multiple alignment on the simulated SIM and the empirical SGS-FULL sequence sets (see Datasets) using MAFFT [54], called consensus using the *cons* method of the EMBOSS package [55], and then cropped the LTR regions, yielding 8500–8700 bp near-full-length HIV-1 genomes. For the analysis of the SS+NGS dataset, we used the Sanger sequences as benchmarking reference.

From the SIM and SGS-FULL sequence sets, we generated in silico Illumina MiSeq paired-end short reads with varying fold coverage (500 and 10,000 reads/base for the SIM and 2000 reads/base for the SGS-FULL datasets), a read length of 250 bp, Phred quality scores (Q) set between 20 and 40, and a mean fragment size of 700 bp using the NGS read simulator ART [56]. We also conducted an additional analysis using 500 reads/base coverage without contamination, with read lengths of 150 bp and a mean fragment size of 500 bp.

We employed Trimmomatic [57] to remove low-quality regions (Q < 20) [52] at the beginning and end of the reads (using a sliding window of 4 bases). Reads shorter than 50 bp were discarded. We used SMALT to map trimmed reads to the benchmarking reference [58], with an exhaustive search for read mapping, and Picard’s *MarkDuplicates* method [59] to remove any duplicate reads. We inferred single-nucleotide polymorphisms and indels from the reference-mapped processed reads using LoFreq [60]. 

#### 2.2.2. Genome Assemblers

A literature search was conducted using Google Scholar up to May 2023 to identify viral genome assemblers that met the following criteria:

1. The assembler must function as a data processing pipeline.
2. It should be open-source.
3. It should either utilize hybrid assembly methods or employ algorithms specifically designed for viral genomes.
4. It must offer a command-line interface.

We evaluated the performance of four selected viral genome assembly pipelines: shiver [34] (v1.4.3, for which we created a “Dockerized” version to enable easy use: dshiver version v1.4.3_1.0), SmaltAlign (v1.1.0) [36], viral-ngs (v1.25.0) [37,61], and V-pipe (v2.99.3) [62]. The first three pipelines are state-of-the-art tools in viral genome assembly, using a combination of de novo and reference-based assembly (hybrid assembly). They either rely on a reference-based ordering of contigs and subsequent imputation of missing regions (shiver and viral-ngs), or on the iterative mapping of reads to the contigs and a reference sequence (SmaltAlign). The fourth pipeline, V-Pipe, uses a high-precision read mapper specifically designed for viral NGS data (ngshmmalign) to map the reads to a user-specified reference sequence. Figure 1 and Table 1 provide a comparative overview of the analyzed pipelines. Parameters were set to default except for the SS+NGS dataset, where certain parameters in the shiver and viral-ngs pipelines had to be adjusted due to short sequence lengths.

We applied the same steps of genome cropping, read mapping, deduplication, and variant calling to the output consensus genomes.

#### 2.2.3. Dshiver: A Containerized Version of the Shiver Pipeline

To enable effortless use of the shiver pipeline [34] (including by non-bioinformaticians), we packed it into a Docker container [63] that allows simple installation (on both Linux-based and Windows operating systems) and convenient access to the major capabilities of shiver, supplemented with an improved command-line interface and detailed documentation aimed at readers with basic computer skills for easy use of the pipeline. Furthermore, we eliminated the Python 2 dependency of shiver by updating all scripts to Python 3, to facilitate continuing support and easier integration of shiver into larger pipelines. We also added an automated drug resistance report based on the analysis of the consensus sequence using the Stanford HIVdb algorithm [64]. We call this modified version dshiver (Dockerized version of shiver, v1.4.3_1.0).

The code and the manual of dshiver are publicly available at https://github.com/hcovlab/dshiver (accessed on 23 November 2024) and a ready-to-use Docker image can be downloaded at https://github.com/orgs/hcovlab/packages/container/package/dshiver (accessed on 23 November 2024).

#### 2.2.4. Evaluation of System Requirements and the Quality of Assembled Genomes

We assessed the performance of each pipeline using QUAST [65], a dedicated tool for the comparison and quality assessment of genome assemblies. We selected metrics that encompass measurements of genome completeness (fraction of genome assembled) and several aspects of genome accuracy, like mismatch (SNP) and indel rates, the fraction of unidentified bases, and the number of misassemblies. Local and global misassemblies were identified using breakpoints, where the left and right flanking sequences overlapped, were separated by a gap (between 200 bp and 1 kbp for local, and greater than 1 kbp for global), or were positioned on a different strand. Furthermore, we evaluated mapping precision (the difference in the rate of mapped and properly paired reads compared to the benchmark) and the accuracy of minority variant calls using precision (ratio of true positives out of all positive predictions), recall (ratio of true positives correctly identified as positives), and F1 scores (harmonic mean of precision and recall).

To compare the runtime and maximum memory usage of each genome assembler, we performed all analyses on the same computer, with a configuration consisting of an Intel(R) Core(TM) i7-7700 CPU @ 3.60GHz processor, 16 GB of system memory, and an Ubuntu 22.04.2 LTS operating system. The measurements of CPU time, CPU usage, and maximum resident set size (maximum physical memory used) were obtained with the GNU “time” command.

All custom scripts, configuration files, empirical datasets, and raw result files used in our analyses are publicly available at https://github.com/hcovlab/ViralNGSBenchmarking (accessed on 23 November 2024).

#### 2.2.5. Statistical Analyses

To measure the diversity of the simulated and empirical quasispecies datasets, we calculated the mean pairwise Hamming distance (the number of corresponding positions at which two sequences differ) of all aligned sequences. Furthermore, we performed a multivariate logistic regression analysis to assess the effects of quasispecies diversity and divergence from the reference on pipeline failure events.

To test whether our main output metrics (genome fraction recovery, mismatch and indel rates, the number of misassemblies and uncalled bases, mapping precision and variant calling precision, recall, and F1 score) differed between genome assemblers in the SIM dataset scenarios, we calculated Wilcoxon signed-rank tests on paired samples with the Benjamini–Hochberg *p*-value correction.

We performed a statistical analysis to investigate the pipeline-specific effects of sample properties and genome assembly quality on downstream analysis steps. First, we identified the order of analysis steps (quasispecies simulation, genome assembly, read mapping, and variant calling), and then we matched each step with our main output metrics. After that, we conducted Spearman correlation tests on all variable pairs for each genome assembler (adjusting *p*-values using the Benjamini–Hochberg method) to reveal statistical associations between benchmarking metrics. Combining the correlation tests with the order of analysis steps enabled us to identify potential causal relationships between variables in adjacent analysis steps (because the order of steps is fixed, correlation between metrics obtained from subsequent analysis steps is likely to indicate the effect of the earlier step on the next step).

## 3. Results

### 3.1. Comparison of In Vivo and In Silico Quasispecies Datasets

We assessed the performance of four genome assemblers—shiver, SmaltAlign, viral-ngs, and V-Pipe—focusing on assembly quality, precision of read mapping and minority variant calling, and computational resource usage across three distinct HIV-1 datasets. The first encompassed 480 in silico HIV-1 quasispecies sequence sets and sequencing reads, spanning five viral (sub)types and exploring two coverage and two contamination scenarios (SIM). The second dataset comprised five samples from five patients, consisting of full-length SGS results combined with simulated sequencing reads (SGS-FULL). The third dataset included 46 NGS datasets from 41 patients, utilizing both Sanger and next-generation sequencing of the HIV-1 *pol* gene (SS+NGS).

**Figure 2:**
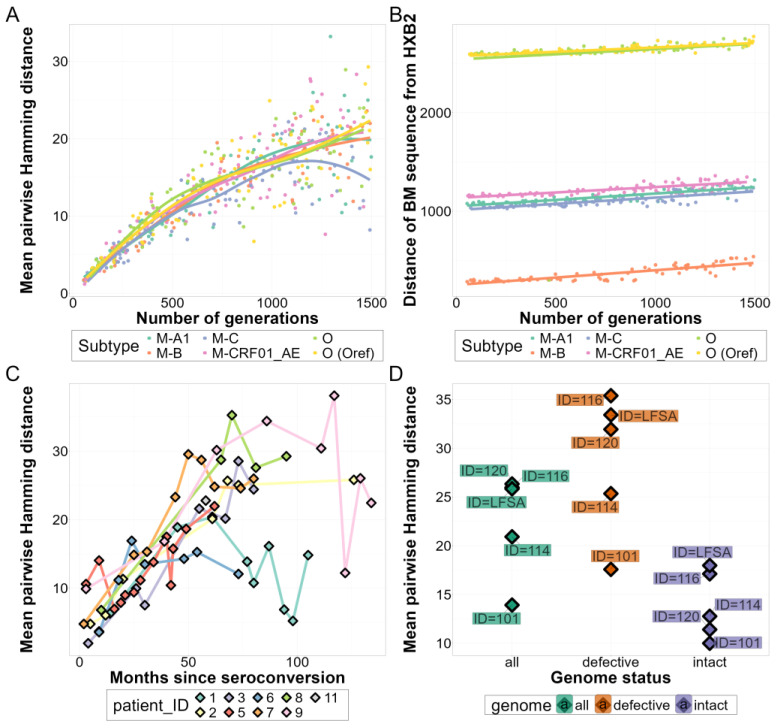
Within-host viral diversity of empirical and simulated quasispecies: Changes in (**A**) quasispecies diversity in the env region and (**B**) whole-genome divergence from reference for all subtypes by the number of generations in the simulated datasets (both measured by their Hamming distances). (**C**) Mean sequence diversity of the *env* gene throughout infection (for samples with 10 or more sequences) reported by Shankarappa et al. [66]. (**D**) Mean average Hamming distances for defective and intact sequence subpopulations and overall populations in the SGS-FULL dataset (limited to the *env* gene). Abbreviations: BM—benchmarking.

The average pairwise Hamming distance of sequences in the quasispecies (referred to as “quasispecies diversity” hereafter) generated by SANTA-SIM (see Figure 2A,B) aligned well with empirical observations (Figure 2C,D). Our results exhibited temporal dynamics, showing low-diversity patterns in simulations with small generation numbers reminiscent of recent infections, and high quasispecies diversity in longer simulations resembling chronic HIV-1 infections. These are consistent with the observations of Shankarappa et al. [66], measuring sequence diversity within the *env* gene (~600 bp) using longitudinal samples from HIV-1-infected patients (see Figure 2C). For the *env* gene, the Hamming distance (at the plateau) in chronic-like in silico sequence sets (IQR: 8.2–17.8) was comparable to the empirical results of Shankarappa et al. [66] (IQR: 10.2–24.7) and was also very similar to the overall diversity of the intact near-full-length sequences in the SGS-FULL dataset (IQR: 11.4–17.1) (Figure 2D). In our simulations, the genetic distance of the quasispecies consensus sequence from the HXB2 reference genome (referred to as “divergence from reference” hereafter) varied strongly with the subtype of the sequence used for the simulations and increased steadily but weakly with the duration of the simulations (Figure 2B).

### 3.2. Quality of Consensus Genome Assemblies and Minority Variant Calls

Three out of the four pipelines exhibited failures (aborted runs). Specifically, viral-ngs showed eight failure events, V-Pipe showed only two, while shiver encountered errors in 25 out of 480 simulations. Quasispecies diversity increased the odds of pipeline failure (logistic regression, Δodds/unit increase = 1.005, *p* = 0.03); however, distance from reference showed no such effect.

**Figure 3:**
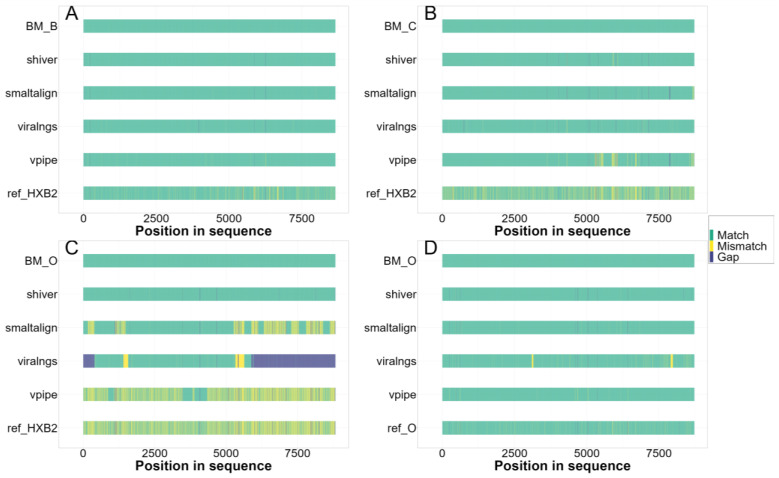
Illustrative comparison of genome assemblies with varying degrees of divergence between the selected simulated samples and the sequence used as a reference in the assembly: Each panel consists of (from top to bottom) the benchmark assembly (“ground truth”) from a simulated sample, assembled consensus genomes generated with the four pipelines, and the reference sequence used in the assembly; color coding illustrates differences (mismatches, indels, and larger gaps) from the benchmark sequence in all cases. (**A**) Group M subtype B sample with a matching HXB2 reference sequence. (**B**) Group M subtype C sample with HXB2 reference sequence. (**C**) Highly divergent group O sample with HXB2 reference sequence. (**D**) Group O sample with a matching group O reference sequence.

We investigated the impact of variations in divergence from the HXB2 reference genome on the reliability of consensus genome assembly (see a set of illustrative cases in Figure 3). In Table 2, we show the comparative performance of the four pipelines with two distinct sets of subtype/reference scenarios: in one set, the fixed-reference pipelines were used with the default HXB2 reference sequence to analyze the non-subtype B datasets; in the other set, the reference sequence used by the fixed-reference pipelines (SmaltAlign, viral-ngs, V-Pipe) was selected to match the subtype/group of the sample (the subtype B scenario from the main analysis, which matches the default HXB2 reference sequence; and the additional group O scenario, where a group O reference sequence was used).

**Figure 4:**
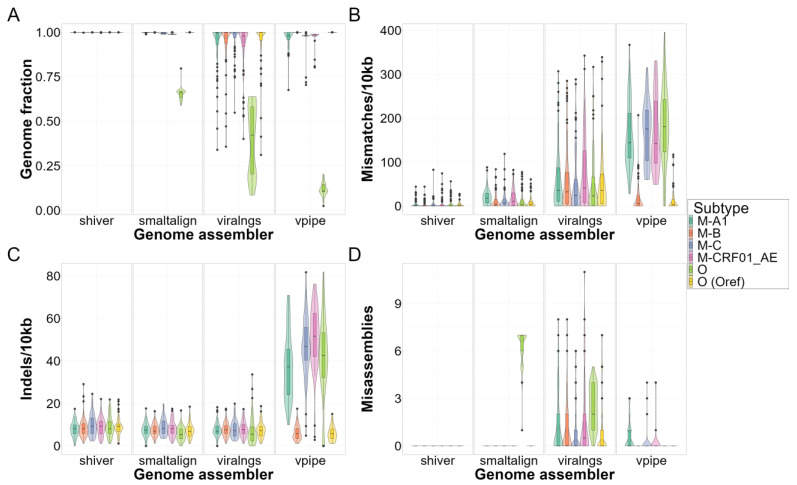
Assembly quality by viral subtype in the SIM dataset: Assemblers are compared based on (**A**) the proportion of recovered positions in the genome, (**B**) the rate of single-nucleotide mismatches per 10 kb, (**C**) the rate of small insertions and deletions (indels) per 10 kb, and (**D**) the number of misassemblies compared to the benchmarking sequence separately for each subtype scenario (see description in the main text). The data shown here include both coverage and contamination scenarios within one subtype.

**Figure 5:**
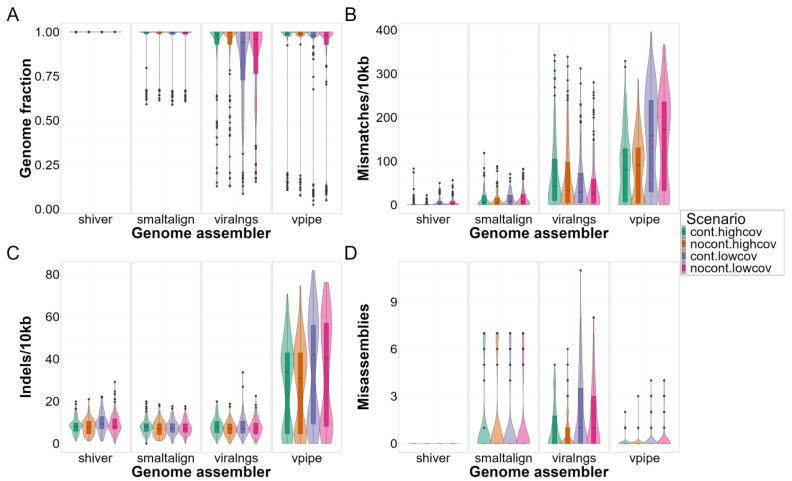
Assembly quality by scenario in the SIM dataset: Assemblers are compared based on (**A**) genome fraction recovery, (**B**) mismatch rates per 10 kb, (**C**) indel rates per 10 kb, and (**D**) the number of misassemblies compared to the benchmarking sequence for the combination of both coverage and contamination scenarios (see description in Materials and Methods). The data shown include simulation results with all virus types. Abbreviations: cont—contaminated, nocont—not contaminated, highcov—high coverage, lowcov—low coverage.

**Figure 6:**
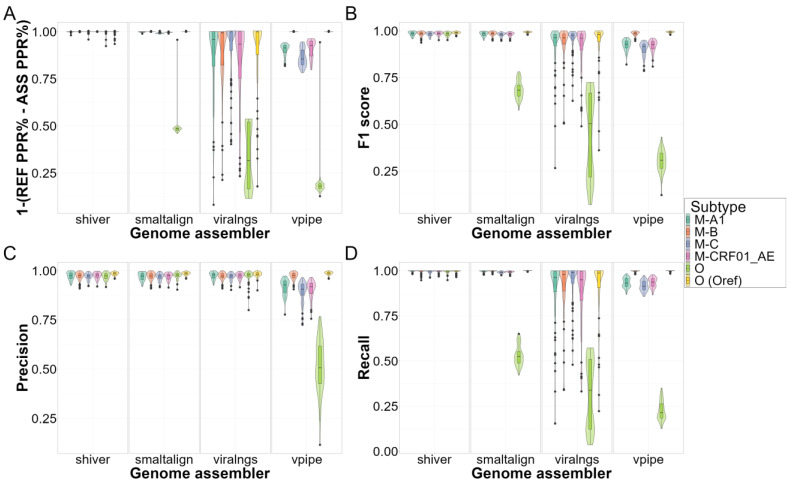
Read mapping and variant calling by viral subtype in the SIM dataset: Assemblers are compared based on (**A**) the difference in the rate of properly paired reads between the assembly and the reference mapping, i.e., read mapping precision, (**B**) the F1 score, (**C**) the precision, and (**D**) the recall of minority variant calling compared to the benchmarking variant set separately for each subtype scenario (see description in the main text). The data shown here include both coverage and contamination scenarios within one subtype. Abbreviations: REF—reference, ASS—assembly, PPR%—percentage of properly paired reads.

The genome assemblers exhibited considerable differences in genome quality (Figure 4 and Figure 5) and subsequent read mapping and variant calling (Figure 6) when the default reference sequence settings were applied. Apart from indel rates, shiver consistently outperformed the other assemblers, achieving complete recovery in nearly all cases (median [Q1–Q3]: 1.0 [1.0–1.0]), and displaying the lowest mismatch rates (1.1 [0.0–4.6] per 10 kb), an absence of misassemblies and variant calling, and high precision (0.98 [0.96–0.99]) and recall (1.0 [0.99–1.0]), irrespective of subtype, coverage, or contamination.

While their pairwise differences were statistically significant (Table 2), SmaltAlign’s overall performance in most scenarios was comparable to that of shiver, with small numerical differences in all metrics (genome fraction recovery: 1.0 [1.0–1.0], mismatch rate: 7.0 [1.2–21.0]/10 kb, indel rate 8.1 [5.8–9.4]/10 kb). However, it exhibited a drop in quality metrics for group O samples, especially concerning genome fraction recovery (group M samples: 1.0 [0.99–1.0] vs. group O samples: 0.66 [0.63–0.67]) and misassemblies (0 [0–0] vs. 7 [6,7]). In regions with low-quality reconstruction, reads did not align well to the assembly (drop in mapping precision from 1.0 [0.93–1.0] to 0.48 [0.48–0.49]), causing minority variants to be unidentified (drop in recall from 0.99 [0.95–1.0] to 0.52 [0.49–0.55]) and in F1 scores from 0.98 [0.94–0.99] to 0.68 [0.65–0.71]).

Genome assemblies generated by viral-ngs exhibited notably low indel rates (7.1 [4.7–9.6]/10 kb) and moderate mismatch rates (31.0 [4.6–85.0]/10 kb) compared to the other pipelines. However, the reconstruction quality in regions with high divergence from the reference was suboptimal, leading to gaps with uncalled bases (2283.6 [1619.5–4074.0]/10 kb for the group O simulations) in the final assembly (mostly near the two ends of the sequence; see also Figure 3) and a subsequent drop in read mapping precision (group M samples: 0.98 [0.81–1.0] vs. group O samples: 0.31 [0.17–0.52]) and variant calling recall (0.97 [0.88–1.0] vs. 0.31 [0.11–0.50]) and F1 scores (0.97 [0.92–0.98] vs. 0.47 [0.20–0.66]).

Finally, V-Pipe showed elevated mismatch (matching reference: 4.0 [0.0–19.7]/10 kb vs. non-matching reference: 162.3 [110.3–225.4]/10 kb) and indel rates (5.8 [3.5–8.1]/10 kb vs. 43.7 [34.4–55.2]/10 kb), a reduction in genome fraction recovery (1.0 [1.0–1.0] vs. 0.98 [0.70–0.99]), an increase in the number of misassemblies (maximum of 0 vs. 7 misassemblies between matching and non-matching scenarios), and lower variant calling recall (1.0 [1.0–1.0] vs. 0.91 [0.60–0.95]) and F1 scores (0.99 [0.99–1.0] vs. 0.91 [0.61–0.93]) in samples with moderately or highly divergent genomes. Unlike the other pipelines, V-Pipe predicted a considerable amount of false positive minority variants, causing a drop in precision (0.98 [0.97–0.99] vs. 0.90 [0.76–0.93]).

In cases where the reference sequence supplied for the fixed-reference pipelines matched the sample, we observed only minor but significant differences between assemblers (comparing them also to shiver; see details in Table 2). Notably, viral-ngs performed significantly worse compared to all other pipelines regarding genome fraction recovery, misassemblies, uncalled bases, and read mapping precision. Additionally, genome sequences produced by V-Pipe showed superior indel rate and variant calling metrics compared to all other genome assemblers when matching reference sequences were used. However, these differences carried through only weakly to later analysis steps. A sensitivity analysis with read lengths of 150 bp instead of 250 bp showed similar results (Figure S1).

We also performed pairwise correlation tests between the output metrics of subsequent analysis steps (quasispecies simulation, genome assembly, read mapping, and variant calling) to identify potential causal relationships between variables (Figures S2 and S3). Our results indicate that the distance from the reference genome primarily affects the completeness of the assemblies (*p* < 0.05 and Spearman correlation coefficient (SCC) < −0.3 for three out of four pipelines), while quasispecies diversity has a greater influence on their correctness (*p* < 0.05 and SCC > 0.3 for three out of four pipelines), both in a pipeline-specific manner. For all pipelines except shiver, both completeness and correctness metrics significantly impact read mapping precision (*p* < 0.05 and SCC < −0.3 or SCC > 0.3), which is a strong predictor of downstream variant calling metrics. For both the SGS-FULL and SS+NGS datasets, assembly quality and variant calling results were nearly equivalent among the assemblers, except for mismatch rates using the SGS-FULL dataset, where shiver (mean mismatch rate: 4.83/10 kb) and SmaltAlign (3.71/10 kb) showed lower rates than viral-ngs (14.75/10 kb) and V-Pipe (13.92/10 kb) (Supplementary Figures S4–S7). 

### 3.3. Computational Resource Use

**Figure 7:**
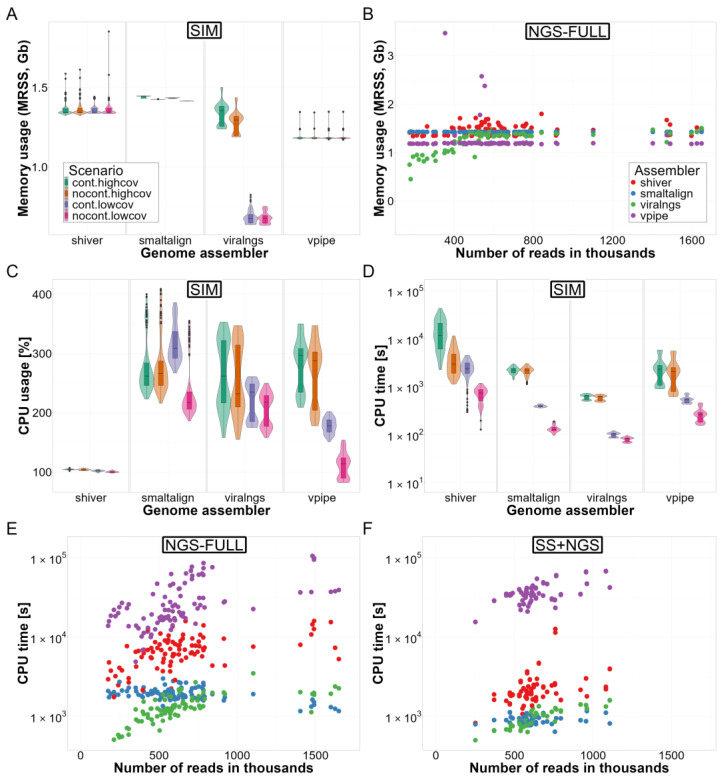
Runtime and memory usage by scenario: Comparison of genome assemblers based on computational needs. Maximum memory usage (MRSS) is compared in the (**A**) SIM and (**B**) NGS-FULL datasets, (**C**) CPU usage (100% equals 1 full CPU core) in the SIM dataset, and CPU time in the (**D**) SIM, (**E**) NGS-FULL, and (**F**) SS+NGS datasets. Panels (**A**), (**C**), and (**D**) stratify results according to coverage and contamination scenarios, while panels (**B**), (**E**), and (**F**) show trends with varying dataset size (number of reads). On panels (**B**), (**D**), (**E**), and (**F**), the benchmarking metric is shown on a log10 scale. Abbreviations: MRSS—maximum resident set size, cont—contaminated, nocont—not contaminated, highcov—high coverage, lowcov—low coverage.

Maximum memory usage was similar across the examined pipelines (maximum resident set size: shiver: 1.35 [1.34–1.37] Gb, SmaltAlign: 1.43 [1.42–1.44] Gb and V-Pipe: 1.18 [1.18–1.18] Gb) except for viral-ngs, which demonstrated lower memory requirements when dealing with low-coverage datasets (high coverage: 1.32 [1.25–1.36] Gb vs. low coverage: 0.68 [0.65–0.70] Gb) (Figure 7A,B). However, we observed substantial variations in CPU time. In the SIM dataset analyses, viral-ngs demonstrated the shortest runtime (CPU time: 265 [81–603] s), closely followed by SmaltAlign (783 [272–2206] s) and V-Pipe (550 [329–1614] s), while shiver required 4–5 times more runtime on average (2473 [876–5497] s) (Figure 7D). The runtime of all genome assemblers was influenced by genome sequencing coverage, and shiver was particularly affected by contaminant reads (contaminated: 3842 [2235–11702] s vs. non-contaminated: 1096 [681–2919] s), mainly because the number of calculations performed by the de novo assembler IVA increases drastically with the presence of contaminant reads (see Supplementary Figure S8). Furthermore, shiver lacks multithreading support (Figure 7C), resulting in even greater differences in elapsed real time compared to viral-ngs and SmaltAlign (Supplementary Figure S9). Finally, in the analyses of empirical read sets, while shiver’s runtime (CPU time: 6831 [5036–8614] s) was still longer compared to viral-ngs (1252 [1019–1667] s) and Smalt-Align (1897 [1728–2133] s), V-Pipe required the longest CPU time (19424 [14790–30828] s) to complete genome assembly (Figure 7E,F).

**Figure 8:**
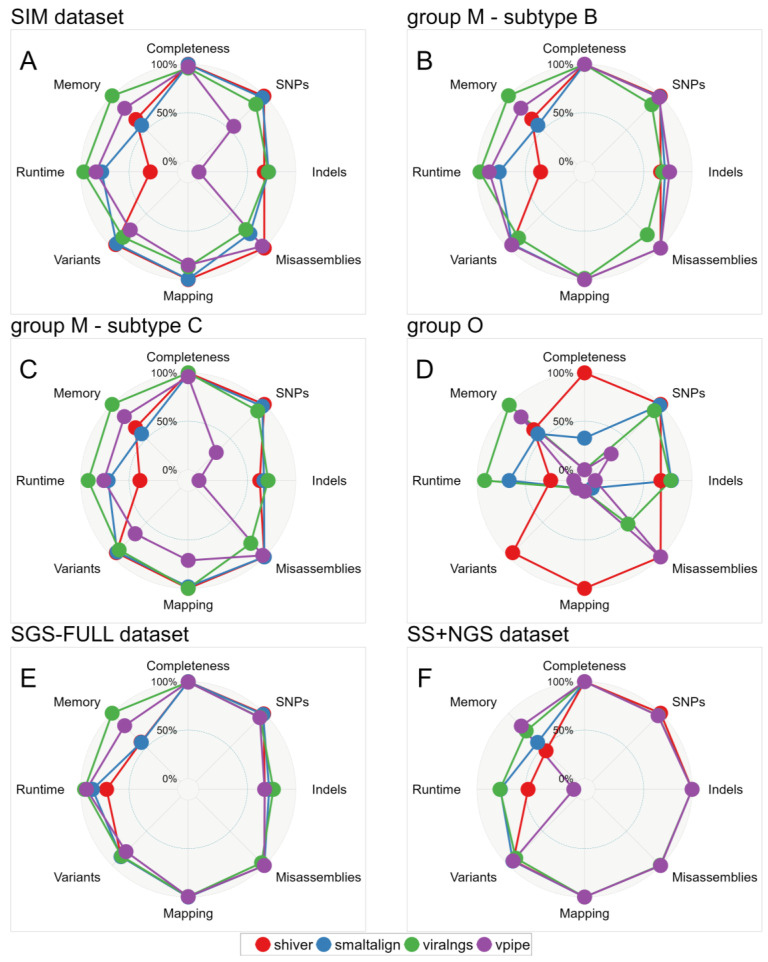
Multidimensional performance of genome assemblers: Benchmarking metrics are compared for (**A**) the SIM dataset—in particular for (**B**) subtype B, (**C**) subtype C, and (**D**) group O results—and for the (**E**) SGS-FULL and (**F**) SS+NGS datasets. For each metric, the relative score between 100% and 0% was calculated using the following threshold values: completeness—100% and 50% median genome fraction recovery; SNPs—0 and 250 median mismatch rate/10 kb; indels—0 and 25 median indel rate/10 kb; misassemblies—0 and 5 mean misassemblies; mapping—100% and 50% median mapping precision; variants—1 and 0.75 median F1 scores; runtime—0 h and 1 h median user time; and memory—1 GB and 2 GB median maximum resident set size.

A summary of all main results from our benchmarking analyses across all three datasets is presented in Figure 8 and Supplementary Figures S10–S12.

### 3.4. Ease of Use

To utilize any of the examined genome assemblers, a basic understanding of the Linux command line and some knowledge of at least one environment management platform (Conda or Docker) are prerequisites. Multiple installation methods are available for all of the pipelines, each accompanied by a comprehensive tutorial outlining the installation process and any dependency requirements. In our experience, due to the complexity of the pipelines, installation tends to require some troubleshooting unless the complete pipeline is available as a fixed containerized version. Among the four pipelines, SmaltAlign, viral-ngs, and V-Pipe are integrated pipelines that do not necessitate the installation of additional bioinformatics tools for basic functionality (V-Pipe offers the option of installing VICUNA for de novo reference construction). In contrast, the original version of shiver requires seven additional tools for genome assembly. However, the containerized dshiver includes all dependencies (other than Docker itself). 

Each pipeline is accompanied by a detailed user guide, including information on available parameters. All of the pipelines, except for SmaltAlign, offer an extensive array of customizable parameters to fine-tune their performance for specific applications, along with some options to modify the workflow structure by selecting specific tools for various stages of the assembly algorithm. This makes SmaltAlign the easiest to use for typical use cases, but less customizable for new scenarios. All of the pipelines are capable of fully automated genome assembly, eliminating the need for additional programming skills or the manual processing of individual samples. Out of the four genome assemblers, shiver offers the most detailed output, including the assembled de novo contigs, position-specific depth and base frequencies, and separate consensus genomes with or without imputation from a reference sequence (and a drug resistance report in dshiver), which are not available in the other pipelines.

The simplified installation process, improved dependency handling, user-friendly automated workflow, and the detailed user guide included with dshiver enable the installation and use of this pipeline with basic computer user skills, on all major operating systems.

In terms of scalability, viral-ngs and V-Pipe stand out as the most convenient pipelines for large-scale analyses, offering mass importing, batch analysis, and advanced multithreading features as integral components of their Snakemake pipelines. In contrast, the other pipelines require some proficiency in shell scripting to perform analyses on multiple samples simultaneously.

## 4. Discussion

Our analyses yielded two main insights. First, all four viral NGS assembly pipelines can produce high-quality genome assemblies when the reference sequence used for the assembly is genetically similar to the sample, which is the typical use case for these tools. Second, we observed relatively poor performance of viral-ngs and V-Pipe on samples with increasing distance from the fixed subtype B (HXB2) reference sequence, and all tools except shiver showed a drop in quality metrics for extremely divergent samples (group O simulations). The main reason behind the robust performance of shiver regardless of viral subtype in the in silico analysis is the tool’s unique feature to select the reference genome that is most similar to the de novo contigs for the imputation of missing regions. In samples with high divergence from the reference, reference-based assembly with a distant reference sequence can result in a biased loss of information due to the inability to map non-matching reads to this reference [42]. As anticipated, the reference-based assembler of V-Pipe and the imputation steps of SmaltAlign and viral-ngs were strongly affected by this problem in the SIM dataset results, especially in genomic regions (e.g., *env* gene) with higher evolutionary distance from the reference.

The consistently reliable results achieved by all assemblers in the empirical analyses can be understood in the light of insights gained from the simulated dataset. The SGS-FULL dataset comprises samples from patients infected with subtype B viral populations only, and the SS+NGS scenario only assesses the quality of the short and high-coverage pol regions of the near-full-length genome assemblies.

Our results indicate that previous analyses of HIV-1 subtype B sequences by other authors have been reliable with all four pipelines, and this is likely to have been the main use case for three of the four pipelines (except for shiver, which has been used for large sequencing efforts in Africa [67,68,69], and which can robustly handle non-subtype B and even non-group M sequences). To be reliably applicable to non-subtype B or non-group M HIV samples, viral-ngs, V-Pipe, and (only for non-group M) SmaltAlign need to be provided with a matched reference sequence, which can also be implemented with automatic selection from a sequence alignment covering the within-species diversity of the virus (as in the shiver pipeline). While it is still common practice to employ the HXB2 sequence as a reference for HIV-1 genome assembly [70,71,72,73,74], selecting a more suitable reference sequence is a viable option even without the modification of the genome assembler pipeline. To guide the selection, the predominant viral subtype can be determined by HIV subtyping tools such as REGA [53] or COMET [75], using either Sanger sequencing data (if available from the same sample) or the result of the first round of genome assembly.

In this study, we compared open-source pipelines that use hybrid genome assembly (shiver, SmaltAlign, viral-ngs) or a reference-based workflow specifically designed for small and highly diverse viral genomes (V-Pipe), and that are readily accessible to users with limited bioinformatics expertise. We excluded pipelines that are only available as a web-based service (like VirAmp [35] or the Genome Detective Platform [76]), as well as proprietary software.

We evaluated complete pipelines, and not the individual bioinformatics tools that are the components of these pipelines. However, since the performance of these components can vary, their selection (for which alternative options are offered in some of the pipelines) might influence the performance of the evaluated pipelines. Previous benchmarking studies for de novo assemblers [77,78], mapping algorithms [34,62,79], and variant calling software [62,78] have demonstrated substantial performance differences, and these studies also highlighted tool-specific characteristics and limitations, which should be considered and possibly compensated for when designing a genome assembly pipeline. For example, IVA (the default assembler in the shiver pipeline) produces large contigs, but sometimes at the expense of genome coverage, whereas SPAdes (default for viral-ngs) generates smaller contigs that cover a larger proportion of the genome [78]. With these settings, shiver may then need to fill in longer missing regions, while viral-ngs will be more dependent on contig filtering, placement, orientation, and refinement. Therefore, tool selection should be viewed in the context of the entire workflow.

The present work is subject to limitations. While each investigated pipeline involves a large number of variable parameters that may influence performance metrics, we used fixed parameter sets, as constraints of time and computational resources restricted the exploration of further combinatorial dimensions in the parameter space. However, expert customization can only improve the performance of the pipelines and, therefore, cannot affect the main finding of good performance by all four pipelines in the key quality metrics. Furthermore, the use of fixed (default) parameterization is a scenario that most closely reflects the use case of NGS analyses by non-specialists.

Due to the scarcity of publicly available near-full-length SGS and paired Sanger and next-generation sequencing datasets, we utilized in silico simulated data in our main analysis. This method offers several advantages, including the availability of a precisely known “ground truth” on the quasispecies analyzed, which allows for more reliable benchmarking comparisons and precise control over sample properties such as diversity, subtype, laboratory contamination, and sequencing depth—factors that can also influence results from experimental datasets. In contrast to experimental data, in silico analyses are not subject to sequencing errors inherent to experimental methods, and they are not constrained by the limited number of single genomes per sample that can be affordably generated, which is typical for SGS datasets. Finally, our complementary analyses on available experimental data yielded mostly consistent results.

We should note that, while sequencing read simulators aim to replicate various characteristics of sequencing data and biases caused by laboratory protocols [80], our chosen tool, ART, like other short-read simulators, has limitations in addressing some factors that may have impacted our analysis. These include biased amplification due to primer mismatches [81], which can cause certain haplotypes to appear more or less frequently in the read data, possibly reducing the performance of all assemblers, along with variations in sequencing depth across different genomic regions, resulting in low-coverage regions, a phenomenon often observed in empirical NGS results of HIV-infected samples [82,83]. The presence of these and other unidentified factors may lead to disparities between in silico and empirical read sets. Such disparities may have contributed to the observed differences in runtime performance between the SIM and the NGS-FULL and SS+NGS datasets. Additionally, we used paired-end read lengths of 2 × 250 bp in our main in silico analysis. While we recognize that shorter read lengths (such as 2 × 150 bp) are still more prevalent in practice, we opted to align with the growing preference for longer read lengths. To also validate the relevance of our main results for shorter read lengths, we analyzed an additional 2 × 150 bp read length scenario, as depicted in Figure S1.

The preprocessing of short-read NGS datasets requires careful consideration. Although our selected read preprocessing tool, Trimmomatic, does not have the ability to remove ambiguous nucleotide positions (Ns) from sequencing reads, we found that these artifacts were largely absent in both our simulated and empirical datasets. For a comprehensive overview of different approaches and recommendations for error correction of NGS datasets, see [84].

Finally, our work focused on the assembly of viruses from short-read sequence data. Assembling datasets generated by different sequencing technologies may require tailored bioinformatics solutions. For instance, in the sequencing and genome assembly of giant viruses with a genome length of approximately 280–2500 kb [85], mate pair sequencing provides additional information on large-scale genome organization, thereby enhancing assembly contiguity and completeness [86,87,88]. In such extreme cases, algorithms used for the assembly of more complex genomes (like bacteria or eukaryotes) might yield more accurate results. Finally, we should note that long-read sequencing technologies mitigate most of the challenges related to the assembly of viral genomes [89]; however, the high per base cost and the technical difficulties related to library preparation and signal processing (leading to high error rates) still limit the applicability of this method in routine viral diagnostics [90].

We employed HIV-1 data as a test case to assess genome assemblers. HIV-1 is one of the most extensively researched and medically significant viruses, demonstrating high levels of diversity both within and between hosts. However, none of the examined pipelines are specifically designed for (or restricted to) HIV-1, and they can be readily adapted to other viruses. Our results emphasize the need to select a matching reference sequence for assembly, especially for other viruses, like the hepatitis C virus, where genetic distances tend to be larger than those observed for HIV [91]. Furthermore, the contaminant filtering feature of shiver may be more relevant if the samples contain phylogenetically closer contaminant reads (and may have to be used cautiously for viruses that tend to pick up unique human genomic fragments, like the hepatitis E virus [92,93]).

In summary, our analysis has addressed a gap in current research by benchmarking state-of-the-art open-source genome assembly pipelines for small viruses. Reassuringly, all four pipelines can perform well when provided with a matching reference sequence, although our results highlight some caveats, specific strengths of individual pipelines, and differences in their practical usability.

## Figures and Tables

**Table 1:** Comparison of the functionalities of the examined pipelines: This table presents information on the available environment management (EM) options and pipeline versions (V) used in this paper, and on whether the pipeline incorporates de novo assembly (DN) and reference-based assembly (RB), reference sequence selection (RS), read quality preprocessing (QP), contamination filtering (CF), or parallel computing (PC) steps. Parallel computing is examined at both the pipeline (usage of multiple cores—first sign) and batch analysis (parallel computing of multiple samples—second sign) levels.

Name	EM	V	DN	RB	RS	QP	CF	PC
**shiver**	-/VirtualBox/Docker ^1^	v1.4.3	+	+	+	+	+	-/-
**SmaltAlign**	Conda	v1.1.0	+	+	-	-	-	+/-
**viral-ngs**	DNAnexus/Conda/Docker/Snakemake	v1.25.0	+	+	-	+	-	+/+
**V-pipe**	Snakemake (Conda)	v2.99.3	-	+	-	+	-	+/+

^1^ The original version of shiver can be installed without an environment management system using an installation script, and it is also available as a VirtualBox image. We have created a Dockerized version (dshiver), described in this paper.

**Table 2:** Comparison of genome assemblers based on quality metrics in the SIM dataset analyses: Quality metrics for genome assemblers were assessed in the analyses that used the SIM dataset, and significant differences (fdr-adjusted *p*-value < 0.05) were determined through Wilcoxon signed-rank tests on paired samples. The result of each pairwise comparison is depicted in three columns: the first (header: acronym of tool 1) shows the number of scenarios where the first tool had significantly higher performance in a genome assembly metric, the second column (header: “X”) shows the number of scenarios with no significant difference, and the third column (header: acronym of tool 2) shows the number of scenarios where the second tool performed significantly better. The performance of the assemblers was ordered based on the count of significant differences observed across all 24 simulated scenarios, with relations categorized as equal (0, =), comparable (1–2, ≈), or differential (3+, >). Abbreviations: X—not significant, SH—shiver, SA—SmaltAlign, VN—viral-ngs, VP—V-Pipe.

Metric	Reference ^1^	Genome Assembler Pairwise Comparisons																	Order	
		SH	X	SA	SH	X	VN	SH	X	VP	SA	X	VN	SA	X	VP	VN	X	VP	
Genome fraction	Matching	0	7	1	6	2	0	0	8	0	5	3	0	0	8	0	0	3	5	SA ≈ SH = VP > VN
	Default	16	0	0	15	1	0	16	0	0	9	7	0	16	0	0	4	12	0	SH > SA > VN > VP
Mismatches	Matching	4	4	0	8	0	0	5	3	0	8	0	0	4	3	1	0	3	5	SH > SA > VP > VN
	Default	15	1	0	16	0	0	16	0	0	16	0	0	16	0	0	15	1	0	SH > SA > VN > VP
Indels	Matching	0	4	4	0	5	3	0	2	6	0	8	0	1	3	4	0	4	4	VP > SA = VN > SH
	Default	1	8	7	0	12	4	16	0	0	0	12	4	16	0	0	16	0	0	VN > SA > SH > VP
Misassemblies	Matching	0	8	0	4	4	0	0	8	0	5	3	0	0	8	0	0	3	5	SA = SH = VP > VN
	Default	4	12	0	14	2	0	3	13	0	10	2	4	3	9	4	0	5	11	SH > VP ≈ SA > VN
Ns	Matching	0	8	0	4	4	0	0	8	0	8	0	0	0	8	0	0	0	8	SA = SH = VP > VN
	Default	0	16	0	10	6	0	0	16	0	16	0	0	0	16	0	0	0	16	SH = SA = VP > VN
Mapping precision	Matching	0	6	2	3	5	0	3	3	2	8	0	0	3	5	0	0	2	6	SA ≈ SH ≈ VP > VN
	Default	12	4	0	9	7	0	14	2	0	10	6	0	16	0	0	6	10	0	SH > SA > VP > VN
Variants F1 score	Matching	0	4	4	3	5	0	0	4	4	8	0	0	1	4	3	0	0	8	VP ≈ SA > SH > VN
	Default	11	5	0	10	6	0	14	2	0	10	6	0	16	0	0	8	8	0	SH > SA > VN > VP
Variants precision	Matching	0	5	3	1	7	0	0	5	3	2	6	0	1	3	4	0	4	4	VP > SA > SH ≈ VN
	Default	7	7	2	0	15	1	14	2	0	0	9	7	16	0	0	16	0	0	VN ≈ SH > SA > VP
Variants recall	Matching	0	3	5	3	5	0	0	5	3	2	6	0	1	3	4	0	4	4	VP > SA > SH > VN
	Default	9	7	0	10	6	0	14	2	0	11	5	0	16	0	0	2	14	0	SH > SA > VN ≈ VP

^1^ Default reference sequences differ between shiver (which uses a customized LANL consensus alignment) and other assemblers (which use HXB2 as a fixed reference). Additional analyses were performed using reference sequences selected to match the subtype of the analyzed sample for the three pipelines with a fixed reference.

## Data Availability

The Sanger sequence data used in this study have been submitted to GenBank at https://www.ncbi.nlm.nih.gov/nucleotide/ (accessed on 23 November 2024) under accession numbers MK213294, MK213306, MK236513, MK236525, MK250657, MK250672, MK250680, PP313557-PP313598, and PP333487-PP333522, and sequencing reads have been submitted to the Sequence Read Archive at https://www.ncbi.nlm.nih.gov/sra (accessed on 23 November 2024) under BioProject accession number PRJNA1078284 and BioSample accession numbers SAMN39993709-SAMN39993754 and have been tagged to be released within 1 year. Until their release, these datasets can be accessed at the GitHub repository of this project (https://github.com/hcovlab/ViralNGSBenchmarking (accessed on 23 November 2024)) to enable full immediate replicability of our studies.

## Supplementary Materials

Supporting information can be downloaded at https://www.mdpi.com/article/10.3390/v16121824/s1, Table S1: Comparison of reverse transcription and nested PCR reaction mixtures; Table S2: Cycling conditions of reverse transcription and nested PCR; Table S3: Primers used in reverse transcription and nested PCR; Figure S1: Assembly and variant calling quality by viral subtype in a simulated dataset with 150 bp read length; Figure S2: Correlation of genome assembly metrics in the SIM dataset; Figure S3: Correlative and potential causal relationships among benchmarking metrics; Figure S4: Assembly quality in the SGS dataset; Figure S5: Read mapping and variant calling in the SGS-FULL dataset; Figure S6: Assembly quality in the SS+NGS dataset; Figure S7: Read mapping and variant calling in the SS+NGS dataset; Figure S8: Sensitivity of shiver (IVA) to the presence of contamination during the analysis of in silico sequencing reads; Figure S9: Comparison of elapsed time between assemblers; Figure S10: Multidimensional performance of genome assemblers in the subtype A1 and CRF01_AE scenarios; Figure S11: Multidimensional performance of genome assemblers in high coverage scenarios; Figure S12: Multidimensional performance of genome assemblers in low coverage scenarios.
