# Supplementary material for: Comparative Evaluation of Open-Source Bioinformatics Pipelines for Full-Length Viral Genome Assembly
Source: Viruses. 2024 Nov 24;16(12):1824. doi: 10.3390/v16121824 (PMC11680378; doi:10.3390/v16121824)
Supplement: Supplementary file 1 [file viruses-16-01824-s001.zip › viruses-3296085-supplementary.pdf]

Title:

Comparative Evaluation of Bioinformatic Pipelines for Full-Length Viral Genome Assembly

Supplementary Materials

Authors:

Levente Zsichla[1,2], Marius Zeeb[3,7], Dávid Fazekas[1,4], Éva Áy[2,5], Dalma Müller[1,2,6], Karin J. Metzner[3,7], Roger Kouyos[3,7], Viktor Müller[1,2]

Supplementary information: Next-generation sequencing protocol 1

Supplementary tables: specifications of the reverse transcription and nested PCR protocol 2

Supplementary figures: Assembly and variant calling quality by viral subtype in a simulated dataset with 2x150bp read length 4

Supplementary figures: Correlation of genome assembly metrics... 5

Supplementary figures: Quality of genome assemblies using the SGS-FULL dataset 8

Supplementary figures: Quality of genome assemblies using the SS+NGS dataset 9

Supplementary figures: contamination sensitivity of de novo assembler IVA 10

Supplementary figures: Comparison of real time requirements of the examined pipelines......... 10

Supplementary figures: Multivariate comparison of pipelines in two subtype scenarios 11

Supplementary figures: Multivariate comparison of pipelines in the combination of coverage and contamination scenarios 12

Supplementary information: A list of studies that used the benchmarked viral genome assembly pipelines 13

Supplementary references 17

Supplementary information: Next-generation sequencing protocol

A next-generation sequencing protocol was developed for the amplification of near full-length HIV-1 genome and short-read sequencing based on previous publications [1]. Viral RNA was extracted from 300 or 280 µl EDTA-treated plasma using NucliSENS miniMAG nucleic acid purification system and specific magnetic extraction reagents (bioMérieux) or QIAamp Viral RNA Mini kit (Qiagen) as per manufacturers' instructions. Purified viral RNA was stored at -80°C or immediately reverse transcribed into cDNA applying a PrimeScript High Fidelity RT-PCR kit (Takara Bio) 2 step RT-PCR protocol with oligo dT or gene specific primers. Nearly full-length HIV-1 genomes were amplified in four overlapping fragments in nested PCR using MyFi Mix (2x) (Meridian Bioscience). Composition of reaction mixtures, thermal cycling conditions and PCR primers applied in reverse transcription and nested-PCR are listed in Table S1-S3. The quality of

amplicons was verified by agarose gel electrophoresis. Four PCR amplicons of each sample were purified using AMPure XP beads (Beckman Coulter) and were pooled in equimolar amounts by using Qubit dsDNA HS Assay kit and Qubit 4 Fluorometer (Invitrogen). NGS library preparation was performed following the manufacturer's protocol of Nextera XT Library Preparation and Nextera XT Index kit v2 (Illumina). All quantification steps were implemented using Qubit dsDNA HS Assay kit during library preparation. The size distribution of libraries was assessed using High Sensitivity D1000 Reagents and ScreenTape on TapeStation 2200 system (Agilent Technologies). Paired-end reads were sequenced with MiSeq Reagent v2 kit (300 cycles) on MiSeq instrument (Illumina). To evaluate the effects of nucleic acid extraction method, presence / absence of carrier RNA (QIAamp Viral RNA Mini kit), reverse transcription with oligo dT / gene specific primers, and one round / two round PCR on sequencing output, parallel testing of selected samples was performed, resulting in 46 NGS datasets from 41 patients.

Supplementary tables: specifications of the reverse transcription and nested PCR protocol

Table S1: Composition of reverse transcription and nested PCR reaction mixtures

Reaction	Reagent	Amount (µl)	Total amount (µl)
Template denaturation and primer annealing (M1)	dNTP Mixture (10 mM each)	1	10
	oligo dT (2,5 µM) / gene specific primer (10 µM)	1	
	template RNA	5	
	RNase-free dH_2O	3	
Reverse transcription	denatured and annealed reaction mixture (M1)	10	20
	5x PrimeScript Buffer	4	
	RNase Inhibitor (40U/µl)	0.5	
	PrimeScript RTase	0.5	
	RNase-free dH_2O	5	
First round PCR	MyFi Mix, 2x	12.5	25
	Forward primer (10 µM)	1	
	Reverse primer (10 µM)	1	
	Nuclease-free dH2O	5.5	
	cDNS	5	
Second round PCR	MyFi Mix, 2x	12.5	25
	Forward primer (10 µM)	1	
	Reverse primer (10 µM)	1	
	Nuclease-free dH2O	8.5	
	First round PCR product	2	

Table S2: Cycling conditions of reverse transcription and nested PCR

Reaction	Temperature (°C)	Time	Cycle
Template denaturation and primer annealing	65	5 min	1
	4	hold	1
Reverse transcription	42	30 min	1
	70	15 min	1
	4	hold	1
First round PCR	95	2 min	1
	95	15 sec	30
	55	15 sec	
	72	3 min	
	72	1.5 min	1
	4	hold	1
Second round PCR	95	2 min	1
	95	15 sec	35
	55	15 sec	
	72	3 min	
	72	1.5 min	1
	4	hold	1

Table S3: Primers used in reverse transcription and nested PCR

Reaction	Amplicon 1		Amplicon 2		Amplicon 3		Amplicon 4	
	Forward	Reverse	Forward	Reverse	Forward	Reverse	Forward	Reverse
Reverse transcription	-	GAG-RT-1R	-	oligo dT/ RT-IN-1R/ Pan-HIV-1_2R	-	oligo dT/ IN-ENV-1R	-	oligo dT/ ENV-NEF-1R
First round PCR	GAG-RT-1F	GAG-RT-1R	RT-IN-1F	RT-IN-1R/ Pan-HIV-1_2R	Pan-HIV-1_3F	IN-ENV-1R	ENV-NEF-1F	ENV-NEF-1R
Second round PCR	GAG-RT-2F	GAG-RT-2R	RT-IN-2F	RT-IN-2R/ Pan-HIV-1_2R	Pan-HIV-1_3F	IN-ENV-2R	ENV-NEF-2F	ENV-NEF-2R
Reference for primers [1,2]								

Supplementary figures: Assembly and variant calling quality by viral subtype in a simulated dataset with 2x150bp read length

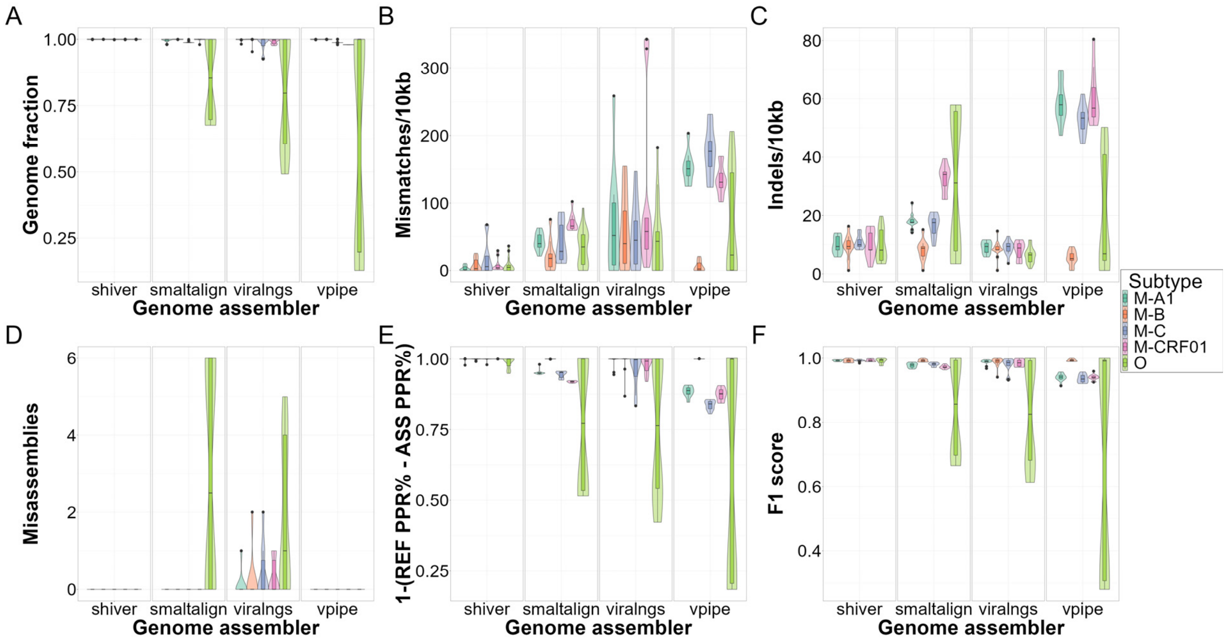

Figure S1: Assembly and variant calling quality by viral subtype in a simulated dataset with 150bp read length. Assemblers are compared based on A) the proportion of recovered positions in the genome, B) the rate of single nucleotide mismatches per 10kb, C) the rate of small insertions and deletions (indels) per 10kb, D) the number of misassemblies, E) the difference in the rate of properly paired reads between the assembly and the reference mapping, and F) the F1 score compared to the benchmarking sequence separately for each subtype scenario (see description in the main text). The data shown here include both coverage and contamination scenarios within one subtype. Abbreviations: REF - reference, ASS - assembly, PPR% - percentage of properly paired reads.

Supplementary analysis: Correlation of genome assembly metrics

We performed an additional analysis to investigate the pipeline-specific effects of sample properties and genome assembly quality on downstream analysis steps (Figure S3). Specifically, we performed pairwise correlation tests between the output metrics of subsequent analysis steps (quasispecies simulation, genome assembly, read mapping, and variant calling) to identify potential causal relationships between variables. These results suggest that divergence from the reference sequence used for assembly had a considerable effect on genome completeness (Figure S2A), which had a negative impact on the precision of read mapping and the recall (and F1 scores) of minority variant detection (Figure S2B). Additionally, quasispecies diversity affected the mismatch and indel rates of genome assemblies (Figure S2C-D), and for V-Pipe and viral-ngs, and these metrics substantially altered subsequent steps of the analysis as well (Figure S2E). In the case of viral-ngs, a greater number of uncalled bases caused a drop in read mapping precision and minority variant calling (Figure S2F), while for V-Pipe, upstream analysis steps affected not only the recall but also the precision of minority variant calling.

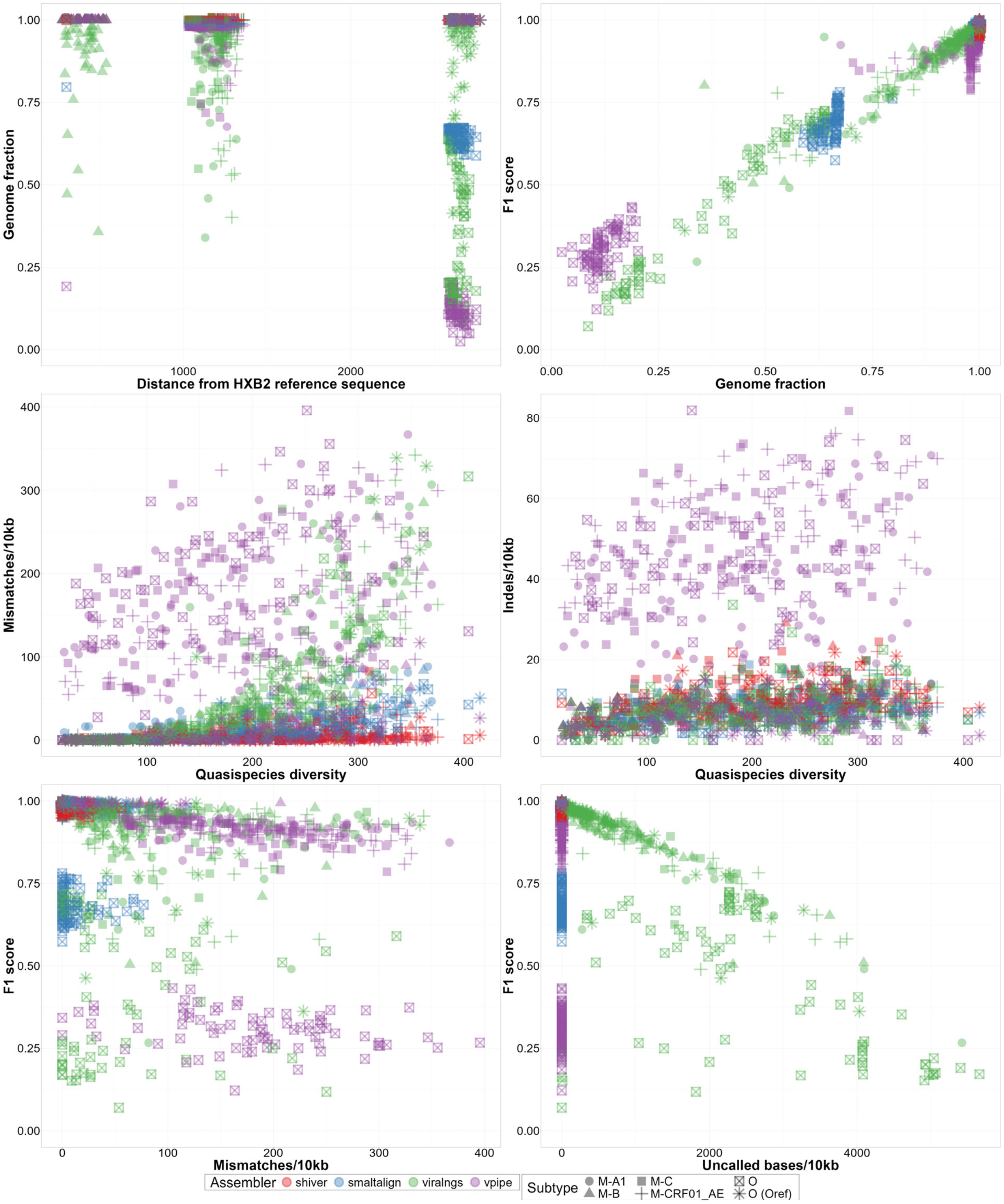

Figure S2: Correlation of genome assembly metrics in the SIM dataset. The panels show the relationship between A) distance from reference and genome fraction, B) genome fraction and variant calling F1 scores, C) quasispecies diversity and mismatch rates, D) quasispecies diversity and indel rates, E) mismatch rates and F1 scores and F) uncalled base rate and F1 scores. Points are grouped according to the genome assembler and the subtype of the sample.

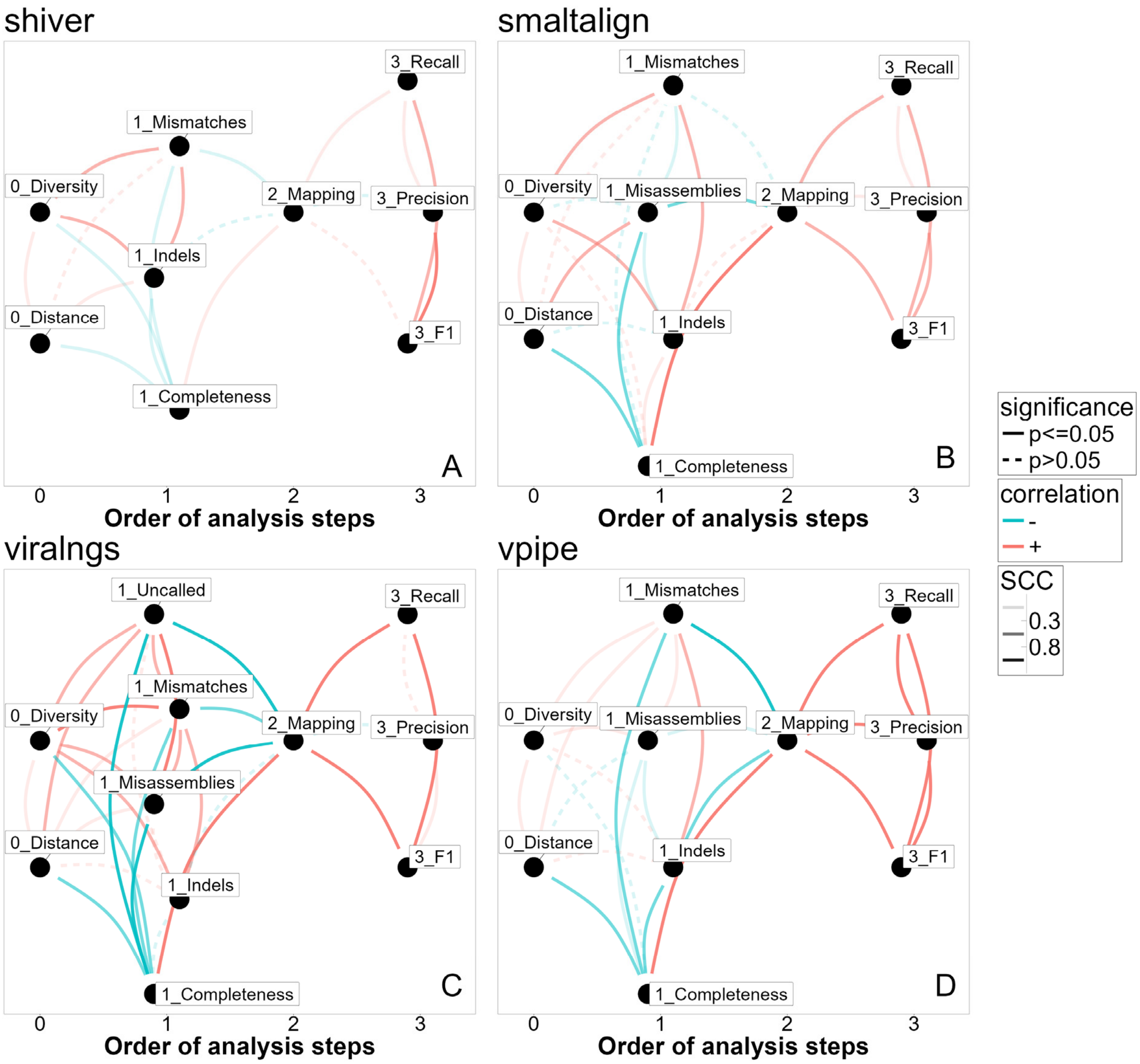

Figure S3: Correlative and potential causal relationships among benchmarking metrics. Undirected acyclic graphs for A) shiver, B) SmaltAlign, C) viral-ngs, and D) V-Pipe illustrating correlative (same analysis step) and potentially causal relationships (adjacent analysis steps) established through Spearman correlation tests between variables. Because the order of steps is fixed, correlation between metrics obtained from subsequent analysis steps is likely to indicate the effect of the earlier step on the next step. The opacity of the edges indicates the strength of the Spearman correlations; the color shows whether the two variables correlate positively or negatively; and solid vs. dashed lines highlight whether the correlation was significant or not. Order of analysis steps: 0 – quasispecies simulation, 1 – genome assembly, 2 – read mapping, 3 – variant calling. The scale of opacity of the edges reflects the absolute value of the correlation coefficient.

Supplementary figures: Quality of genome assemblies using the SGS-FULL dataset

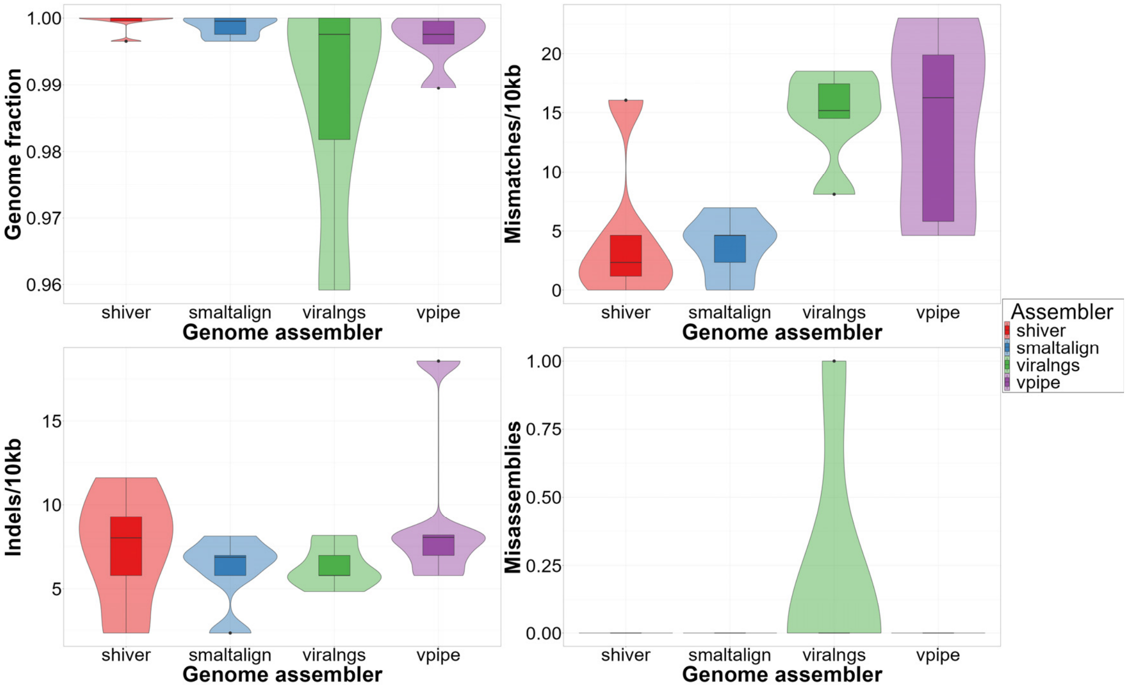

Figure S4: Assembly quality in the SGS dataset. Assemblers are compared based on A) the proportion of recovered positions in the genome, B) the rate of single nucleotide mismatches, C) the rate of small insertions and deletions (indels), and D) the number of misassemblies compared to the benchmarking sequence.

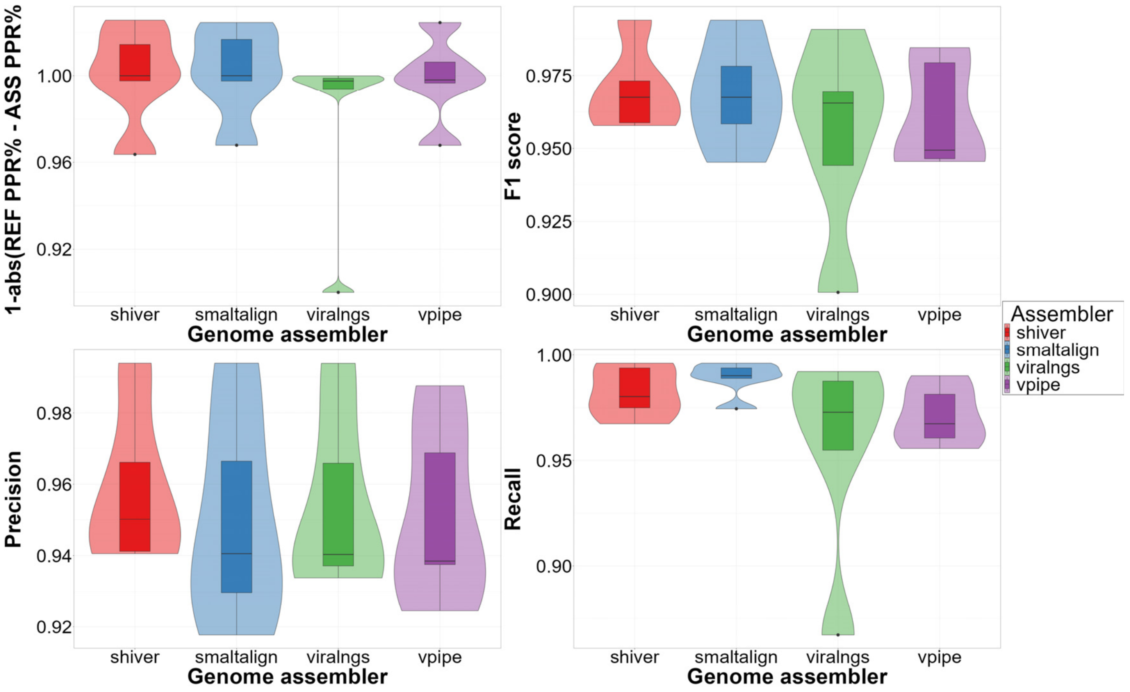

Figure S5: Read mapping and variant calling in the SGS-FULL dataset. Assemblers are compared based on A) the difference in the rate of properly paired reads between the assembly and the reference mapping, and B) the F1 score, C) the precision and D) the recall of minority variant calling compared to the benchmarking variant set. Abbreviations: REF - reference, ASS - assembly, PPR% - percentage of properly paired reads.

Supplementary figures: Quality of genome assemblies using the SS+NGS dataset

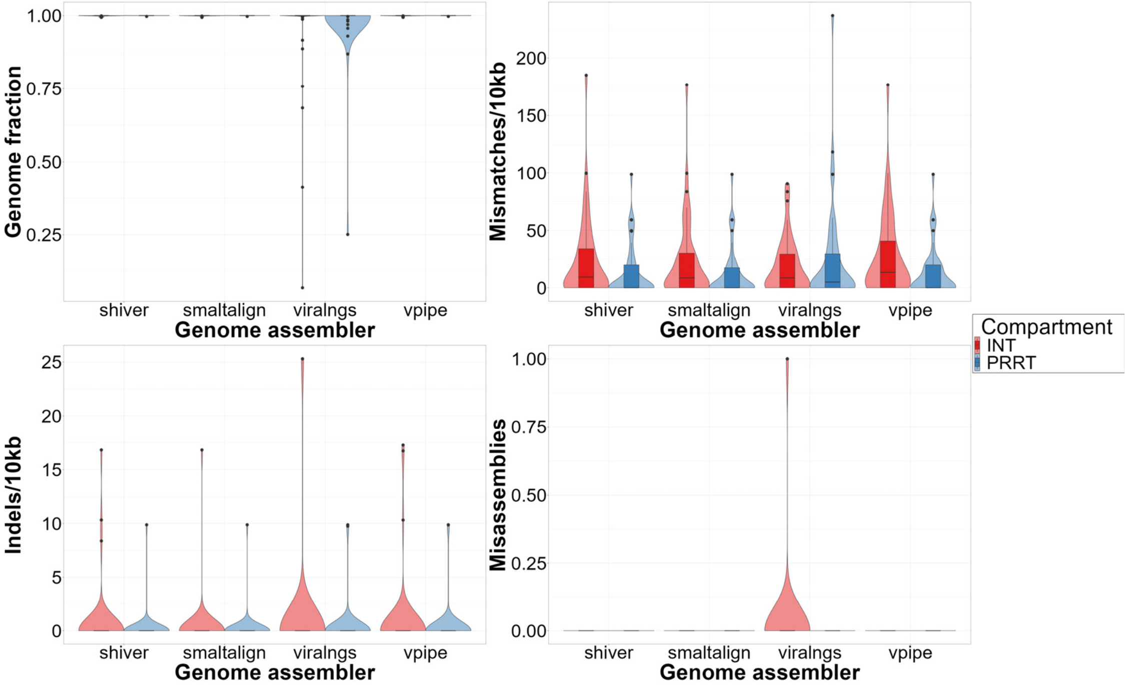

Figure S6: Assembly quality in the SS+NGS dataset. Assemblers are compared based on A) the proportion of recovered positions in the genome, B) the rate of single nucleotide mismatches, C) the rate of small insertions and deletions (indels), and D) the number of misassemblies compared to the benchmarking sequence.

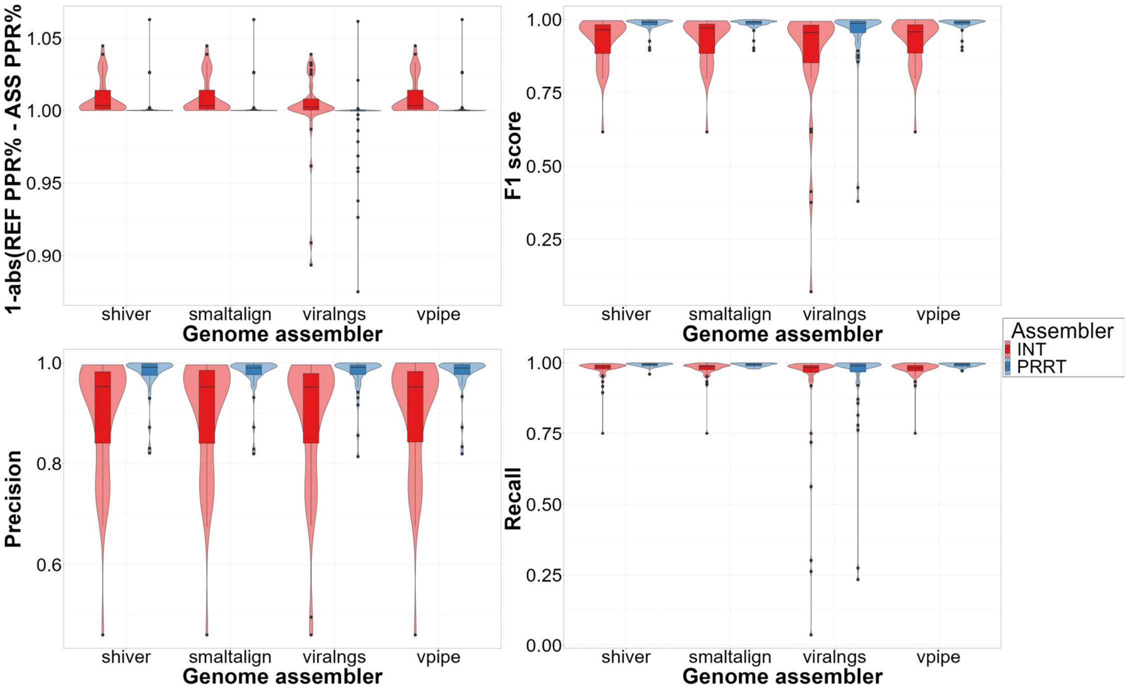

Figure S7: Read mapping and variant calling in the SS+NGS dataset. Assemblers are compared based on A) the difference in the rate of properly paired reads between the assembly and the reference mapping, and B) the F1 score, C) the precision and D) the recall of minority variant calling compared to the benchmarking variant set. Abbreviations: REF - reference, ASS - assembly, PPR% - percentage of properly paired reads.

Supplementary figures: contamination sensitivity of de novo assembler IVA

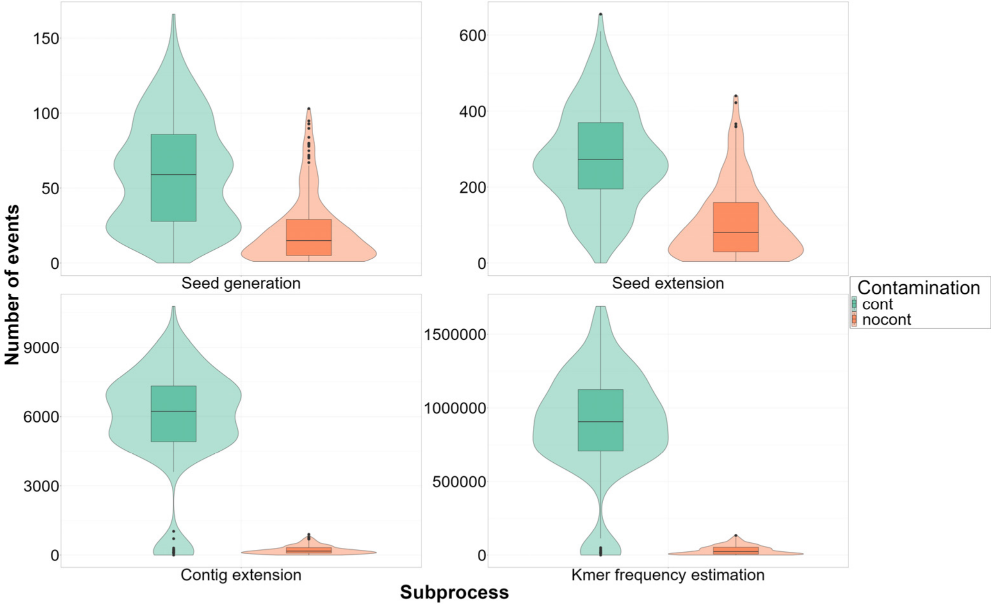

Figure S8: Sensitivity of shiver (IVA) to the presence of contamination during the analysis of *in silico* sequencing reads (SIM dataset). The number of algorithmic steps is considerably higher for contaminated samples compared to not contaminated ones. The differences increase with subsequent steps of the contig generation process (seed generation, seed extension and contig extension) and indicate substantially more computations performed during analysis overall (reflected by the number of k-mer counting steps by KMC as elementary steps of the algorithm).

Supplementary figures: Comparison of real time requirements of the examined pipelines

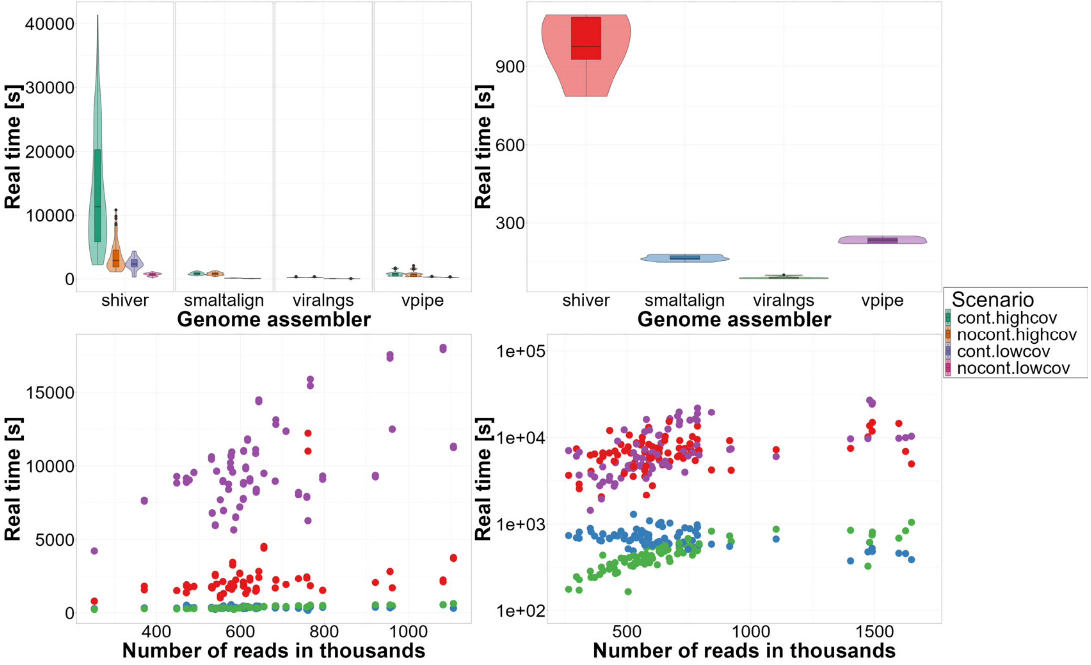

Figure S9: Comparison of elapsed time between assemblers. Comparison of genome assemblers based on runtime (real or elapsed time) in the A) SIM, B) SGS-FULL, C) SS+NGS and D) NGS-FULL datasets. Panel A stratifies results according to coverage and contamination scenarios and panels C and D show trends with varying dataset size (number of reads). Abbreviations: cont – contaminated, nocont – not contaminated, highcov – high coverage, lowcov – low coverage.

Supplementary figures: Multivariate comparison of pipelines in two subtype scenarios

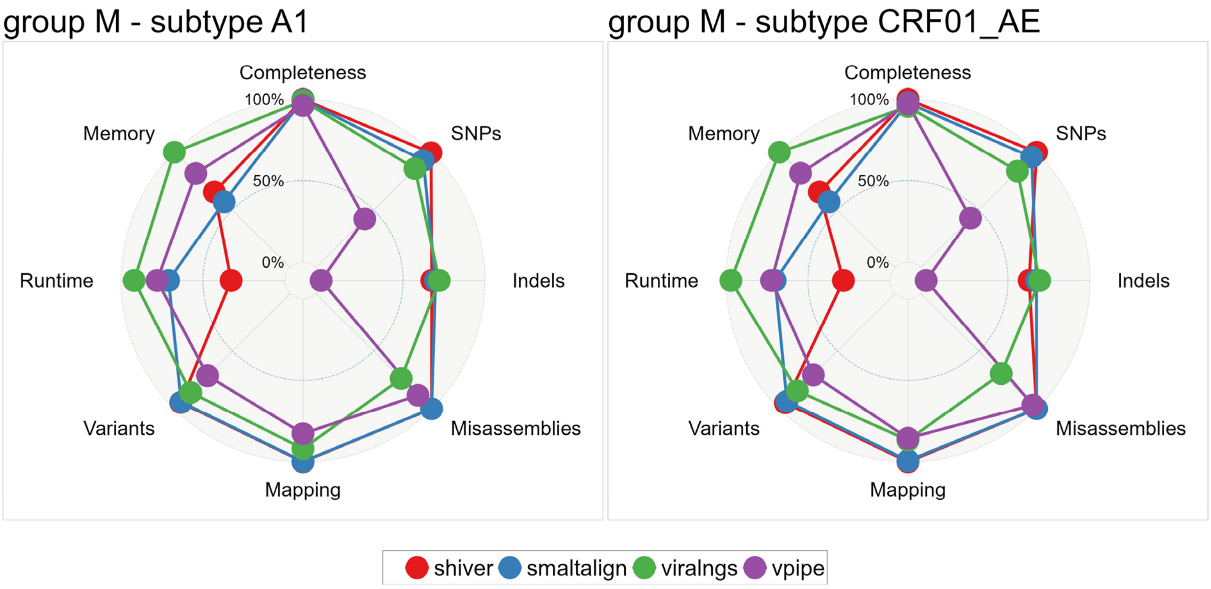

Figure S10: **Multidimensional performance of genome assemblers in the subtype A1 and CRF01_AE scenarios.** Benchmarking metrics are compared for A) the subtype A and B) for the CRF01_AE scenarios of the SIM dataset. For each metric the relative score between 100% and 0% is calculated using the following threshold values: Completeness – 100% and 50% median genome fraction, SNPs – 0 and 250 median mismatch rate/10kb, Indels – 0 and 25 median indel rate/10kb, Misassemblies – 0 and 5 mean misassemblies, Mapping – 100% and 50% median mapping precision, Variants – 1 and 0.75 median F1 scores, Runtime – 0h and 1h median user time and Memory – 1GB and 2GB median maximum resident set size.

Supplementary figures: Multivariate comparison of pipelines in the combination of coverage and contamination scenarios

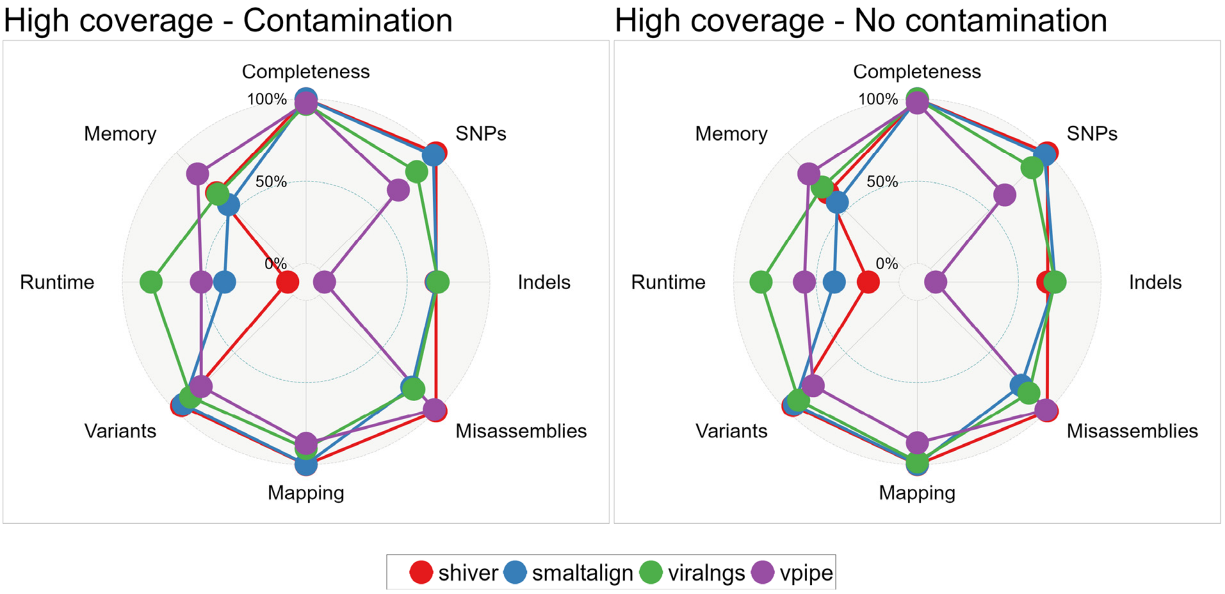

Figure S11: **Multidimensional performance of genome assemblers in high coverage scenarios.** Benchmarking metrics are compared for A) the high coverage – contamination and B) for the high coverage – no contamination scenarios of the SIM dataset. For an explanation on the scaling of the different axes see the figure caption of Figure S9.

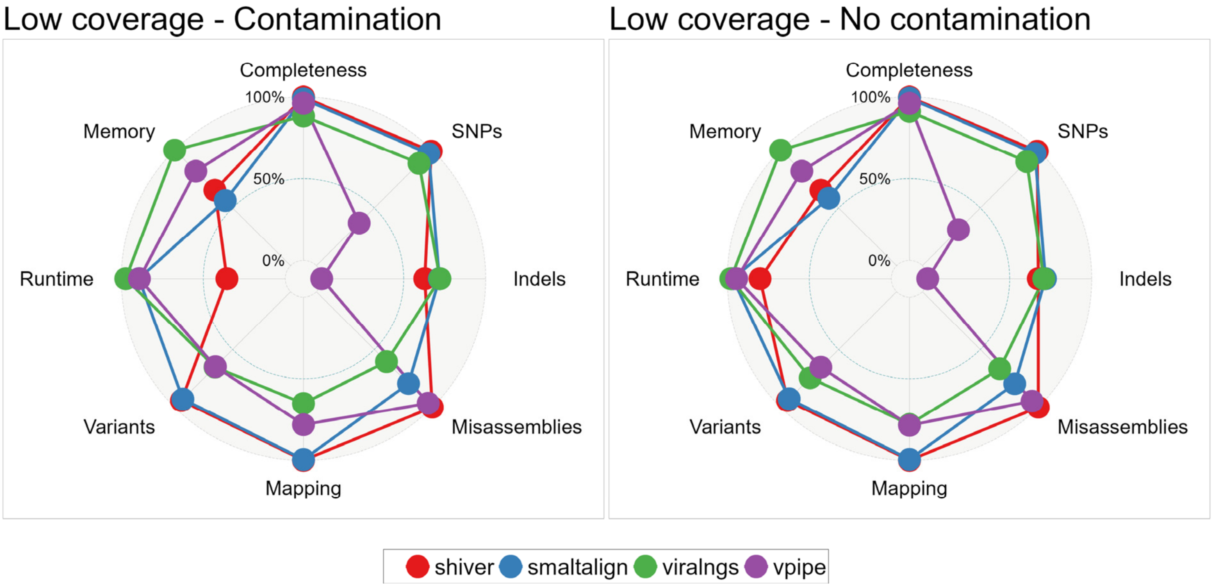

Figure S12: **Multidimensional performance of genome assemblers in low coverage scenarios.** Benchmarking metrics are compared for A) the low coverage – contamination and B) for the low coverage – no contamination scenarios of the SIM dataset. For an explanation on the scaling of the different axes see the figure caption of Figure S9.

Supplementary information: A list of studies that used the benchmarked viral genome assembly pipelines

SmaltAlign

1. Zeeb M, Frischknecht P, Huber M, et al. Genetic diversity from proviral DNA as a proxy for time since HIV-1 infection. The Journal of Infectious Diseases 2024; jiae149
2. Tschumi F, Schmutz S, Kufner V, et al. Meningitis and epididymitis caused by Toscana virus infection imported to Switzerland diagnosed by metagenomic sequencing: a case report. BMC Infectious Diseases 2019; 19:591
3. Weissberg D, Böni J, Rampini SK, et al. Does respiratory co-infection facilitate dispersal of SARS-CoV-2? investigation of a super-spreading event in an open-space office. Antimicrob Resist Infect Control 2020; 9:191
4. Balakrishna S, Loosli T, Zaheri M, et al. Frequency matters: comparison of drug resistance mutation detection by Sanger and next-generation sequencing in HIV-1. J Antimicrob Chemother 2023; 78:656–664
5. Salazar-Vizcaya L, Kouyos RD, Metzner KJ, et al. Changing Trends in International Versus Domestic HCV Transmission in HIV-Positive Men Who Have Sex With Men: A Perspective for the Direct-Acting Antiviral Scale-Up Era. The Journal of Infectious Diseases 2019; 220:91–99
6. Jörimann L, Tschumi J, Zeeb M, et al. Absence of Proviral Human Immunodeficiency Virus (HIV) Type 1 Evolution in Early-Treated Individuals With HIV Switching to Dolutegravir Monotherapy During 48 Weeks. J Infect Dis 2023; 228:907–918
7. Wegner F, Roloff T, Huber M, et al. External Quality Assessment of SARS-CoV-2 Sequencing: an ESGMD-SSM Pilot Trial across 15 European Laboratories. Journal of Clinical Microbiology 2022; 60:e01698-21
8. Marconato M, Abela IA, Hauser A, et al. Antibodies from convalescent plasma promote SARS-CoV-2 clearance in individuals with and without endogenous antibody response. J Clin Invest 132:e158190
9. Pohl MO, Busnadiego I, Kufner V, et al. SARS-CoV-2 variants reveal features critical for replication in primary human cells. PLoS Biol 2021; 19:e3001006

viral-ngs

10. Siddle Katherine J., Eromon Philomena, Barnes Kayla G., et al. Genomic Analysis of Lassa Virus during an Increase in Cases in Nigeria in 2018. New England Journal of Medicine 2018; 379:1745–1753
11. Ajogbasile FV, Oguzie JU, Oluniyi PE, et al. Real-time Metagenomic Analysis of Undiagnosed Fever Cases Unveils a Yellow Fever Outbreak in Edo State, Nigeria. Sci Rep 2020; 10:3180
12. Oguzie JU, Nwangwu UC, Oluniyi PE, et al. Metagenomic sequencing characterizes a wide diversity of viruses in field mosquito samples in Nigeria. Sci Rep 2022; 12:7616
13. Ndiaye T, Sy M, Gaye A, et al. Molecular epidemiology of Plasmodium falciparum by multiplexed amplicon deep sequencing in Senegal. Malar J 2020; 19:403
14. Gaye A, Ndiaye T, Sy M, et al. Genomic investigation of a dengue virus outbreak in Thiès, Senegal, in 2018. Sci Rep 2021; 11:10321
15. Ash KT, Alamilla I, Li Y, et al. Coding-Complete Genome Sequence of a SARS-CoV-2 Variant Obtained from Raw Sewage at the University of Tennessee-Knoxville Campus. Microbiol Resour Announc 2021; 10:e0104921
16. Park DJ, Dudas G, Wohl S, et al. Ebola Virus Epidemiology, Transmission, and Evolution during Seven Months in Sierra Leone. Cell 2015; 161:1516–1526
17. Metsky HC, Matranga CB, Wohl S, et al. Zika virus evolution and spread in the Americas. Nature 2017; 546:411–415

18. Normandin E, Solomon IH, Zamirpour S, et al. Powassan Virus Neuropathology and Genomic Diversity in Patients With Fatal Encephalitis. Open Forum Infect Dis 2020; 7:ofaa392
19. Babiker A, Bradley HL, Stittleburg VD, et al. Metagenomic Sequencing To Detect Respiratory Viruses in Persons under Investigation for COVID-19. J Clin Microbiol 2020; 59:e02142-20
20. Olawoye IB, Oluniyi PE, Oguzie JU, et al. Emergence and spread of two SARS-CoV-2 variants of interest in Nigeria. Nat Commun 2023; 14:811
21. Oguzie JU, Petros BA, Oluniyi PE, et al. Metagenomic surveillance uncovers diverse and novel viral taxa in febrile patients from Nigeria. Nat Commun 2023; 14:4693
22. Nyakarahuka L, Whitmer S, Klena J, et al. Detection of Sporadic Outbreaks of Rift Valley Fever in Uganda through the National Viral Hemorrhagic Fever Surveillance System, 2017–2020. Am J Trop Med Hyg 2023; 108:995–1002
23. Normandin E, Holroyd KB, Collens SI, et al. Intrathecal inflammatory responses in the absence of SARS-CoV-2 nucleic acid in the CSF of COVID-19 hospitalized patients. J Neurol Sci 2021; 430:120023
24. Jaeger AS, Murrieta RA, Goren LR, et al. Zika viruses of African and Asian lineages cause fetal harm in a mouse model of vertical transmission. PLoS Negl Trop Dis 2019; 13:e0007343
25. Folarin OA, Ehichioya D, Schaffner SF, et al. Ebola Virus Epidemiology and Evolution in Nigeria. J Infect Dis 2016; 214:S102–S109
26. Wohl S, Metsky HC, Schaffner SF, et al. Combining genomics and epidemiology to track mumps virus transmission in the United States. PLOS Biology 2020; 18:e3000611
27. Whitmer SLM, Lo MK, Sazzad HMS, et al. Inference of Nipah virus evolution, 1999–2015. Virus Evol 2020; 7:veaa062
28. Langsjoen RM, Key A, Shariatzadeh N, et al. Eastern Equine Encephalitis Virus Diversity in Massachusetts Patients, 1938–2020. Am J Trop Med Hyg 2023; 109:387–396
29. Aliota MT, Dudley DM, Newman CM, et al. Heterologous Protection against Asian Zika Virus Challenge in Rhesus Macaques. PLoS Negl Trop Dis 2016; 10:e0005168
30. Tomkins-Tinch CH, Daly JS, Gladden-Young A, et al. SARS-CoV-2 Reinfection in a Liver Transplant Recipient. Ann Intern Med 2021; L21-0108
31. Levine ZC, Sene A, Mkandawire W, et al. Improving diagnosis of non-malarial fevers in Senegal: Borrelia and the contribution of tick-borne bacteria. medRxiv 2023; 2023.08.24.23294564
32. Normandin E, Valizadeh N, Rudmann EA, et al. Neuropathological features of SARS-CoV-2 delta and omicron variants. J Neuropathol Exp Neurol 2023; 82:283–295
33. Piantadosi A, Freije CA, Gosmann C, et al. Metagenomic Sequencing of HIV-1 in the Blood and Female Genital Tract Reveals Little Quasispecies Diversity during Acute Infection. J Virol 2019; 93:e00804-18
34. Bohm EK, Vangorder-Braid JT, Jaeger AS, et al. Zika Virus Infection of Pregnant Ifnar1−/− Mice Triggers Strain-Specific Differences in Fetal Outcomes. J Virol 95:e00818-21
35. Levine ZC, Sene A, Mkandawire W, et al. Investigating the etiologies of non-malarial febrile illness in Senegal using metagenomic sequencing. Nat Commun 2024; 15:747
36. Jaeger AS, Weiler AM, Moriarty RV, et al. Spondweni virus causes fetal harm in Ifnar1−/− mice and is transmitted by Aedes aegypti mosquitoes. Virology 2020; 547:35–46
37. Uwanibe JN, Kayode TA, Oluniyi PE, et al. The Prevalence of Undiagnosed Salmonella enterica Serovar Typhi in Healthy School-Aged Children in Osun State, Nigeria. Pathogens 2023; 12:594
38. Happi AN, Ogunsanya OA, Ayinla AO, et al. Lassa virus in novel hosts: insights into the epidemiology of lassa virus infections in southern Nigeria. Emerg Microbes Infect 13:2294859
39. Piantadosi A, Mukerji SS, Ye S, et al. Enhanced Virus Detection and Metagenomic Sequencing in Patients with Meningitis and Encephalitis. mBio 12:e01143-21
40. Rodamilans B, Oliveros JC, San León D, et al. sRNA Analysis Evidenced the Involvement of Different Plant Viruses in the Activation of RNA Silencing-Related Genes and the Defensive Response Against Plum pox virus of 'GF305' Peach Grafted with 'Garrigues' Almond. Phytopathology® 2022; 112:2012–2021

41. Knust B, Brown S, de St. Maurice A, et al. Seoul Virus Infection and Spread in United States Home-Based Ratteries: Rat and Human Testing Results From a Multistate Outbreak Investigation. The Journal of Infectious Diseases 2020; 222:1311–1319
42. Shoemaker TR, Nyakarahuka L, Balinandi S, et al. First Laboratory-Confirmed Outbreak of Human and Animal Rift Valley Fever Virus in Uganda in 48 Years. Am J Trop Med Hyg 2019; 100:659–671
43. Otieno JR, Kamau EM, Oketch JW, et al. Whole genome analysis of local Kenyan and global sequences unravels the epidemiological and molecular evolutionary dynamics of RSV genotype ON1 strains. Virus Evol 2018; 4:vey027
44. McMinn RJ, Langsjoen RM, Bombin A, et al. Phylodynamics of deer tick virus in North America. Virus Evol 2023; 9:vead008

V-Pipe

45. Zhakparov D, Quirin Y, Xiao Y, et al. Sequencing of SARS-CoV-2 RNA Fragments in Wastewater Detects the Spread of New Variants during Major Events. Microorganisms 2023; 11:2660
46. Jahn K, Dreifuss D, Topolsky I, et al. Early detection and surveillance of SARS-CoV-2 genomic variants in wastewater using COJAC. Nat Microbiol 2022; 7:1151–1160
47. Francisco I, Bailey S, Bautista T, et al. Detection of Velogenic Avian Paramyxoviruses in Rock Doves in New York City, New York. Microbiol Spectr 10:e02061-21
48. Iketani S, Mohri H, Culbertson B, et al. Multiple pathways for SARS-CoV-2 resistance to nirmatrelvir. Nature 2023; 613:558–564
49. Cancela F, Ramos N, Smyth DS, et al. Wastewater surveillance of SARS-CoV-2 genomic populations on a country-wide scale through targeted sequencing. PLoS One 2023; 18:e0284483
50. Smyth DS, Trujillo M, Gregory DA, et al. Tracking cryptic SARS-CoV-2 lineages detected in NYC wastewater. Nat Commun 2022; 13:635
51. Chan T, Ginders J, Kuhlmeier E, et al. Detection of SARS-CoV-2 RNA in a Zoo-Kept Red Fox (Vulpes vulpes). Viruses 2024; 16:521
52. Dudas G, Hong SL, Potter BI, et al. Emergence and spread of SARS-CoV-2 lineage B.1.620 with variant of concern-like mutations and deletions. Nat Commun 2021; 12:5769
53. Nadeau SA, Vaughan TG, Beckmann C, et al. Swiss public health measures associated with reduced SARS-CoV-2 transmission using genome data. Sci Transl Med 14:eabn7979
54. Bagutti C, Hug MA, Heim P, et al. Wastewater monitoring of SARS-CoV-2 shows high correlation with COVID-19 case numbers and allowed early detection of the first confirmed B.1.1.529 infection in Switzerland: results of an observational surveillance study. Swiss Medical Weekly 2022; 152:w30202–w30202
55. Kuhlmeier E, Chan T, Agüí CV, et al. Detection and Molecular Characterization of the SARS-CoV-2 Delta Variant and the Specific Immune Response in Companion Animals in Switzerland. Viruses 2023; 15:245
56. Lezcano OM, Fuhrmann L, Ramakrishnan G, et al. Parallel evolution and enhanced virulence upon in vivo passage of an RNA virus in Drosophila melanogaster. Virus Evolution 2023; 9:vead074

shiver

57. Emary KRW, Golubchik T, Aley PK, et al. Efficacy of ChAdOx1 nCoV-19 (AZD1222) vaccine against SARS-CoV-2 variant of concern 202012/01 (B.1.1.7): an exploratory analysis of a randomised controlled trial. The Lancet 2021; 397:1351–1362
58. Lythgoe KA, Hall M, Ferretti L, et al. SARS-CoV-2 within-host diversity and transmission. Science 2021; 372:eabg0821
59. Clark IC, Mudvari P, Thaploo S, et al. HIV silencing and cell survival signatures in infected T cell reservoirs. Nature 2023; 614:318–325

60. Wymant C, Bezemer D, Blanquart F, et al. A highly virulent variant of HIV-1 circulating in the Netherlands. Science 2022; 375:540–545
61. Clemens SAC, Folegatti PM, Emary KRW, et al. Efficacy of ChAdOx1 nCoV-19 (AZD1222) vaccine against SARS-CoV-2 lineages circulating in Brazil. Nat Commun 2021; 12:5861
62. Lythgoe KA, Golubchik T, Hall M, et al. Lineage replacement and evolution captured by 3 years of the United Kingdom Coronavirus (COVID-19) Infection Survey. Proceedings of the Royal Society B: Biological Sciences 2023; 290:20231284
63. Bonsall D, Golubchik T, de Cesare M, et al. A Comprehensive Genomics Solution for HIV Surveillance and Clinical Monitoring in Low-Income Settings. Journal of Clinical Microbiology 2020; 58:10.1128/jcm.00382-20
64. Fryer HR, Golubchik T, Hall M, et al. Viral burden is associated with age, vaccination, and viral variant in a population-representative study of SARS-CoV-2 that accounts for time-since-infection-related sampling bias. PLOS Pathogens 2023; 19:e1011461
65. Lin G-L, Drysdale SB, Snape MD, et al. Distinct patterns of within-host virus populations between two subgroups of human respiratory syncytial virus. Nat Commun 2021; 12:5125
66. Ratmann O, Grabowski MK, Hall M, et al. Inferring HIV-1 transmission networks and sources of epidemic spread in Africa with deep-sequence phylogenetic analysis. Nat Commun 2019; 10:1411
67. Magosi LE, Zhang Y, Golubchik T, et al. Deep-sequence phylogenetics to quantify patterns of HIV transmission in the context of a universal testing and treatment trial – BCPP/Ya Tsie trial. eLife 2022; 11:e72657
68. Monod M, Brizzi A, Galiwango RM, et al. Longitudinal population-level HIV epidemiologic and genomic surveillance highlights growing gender disparity of HIV transmission in Uganda. Nat Microbiol 2024; 9:35–54
69. Lin G-L, Drysdale SB, Snape MD, et al. Targeted metagenomics reveals association between severity and pathogen co-detection in infants with respiratory syncytial virus. Nat Commun 2024; 15:2379
70. Ghafari M, Hall M, Golubchik T, et al. Prevalence of persistent SARS-CoV-2 in a large community surveillance study. Nature 2024; 626:1094–1101
71. Zhang Y, Wymant C, Laeyendecker O, et al. Evaluation of Phylogenetic Methods for Inferring the Direction of Human Immunodeficiency Virus (HIV) Transmission: HIV Prevention Trials Network (HPTN) 052. Clinical Infectious Diseases 2021; 72:30–37
72. Zhao L, Hall M, de Cesare M, et al. The mutational spectrum of SARS-CoV-2 genomic and antigenomic RNA. Proceedings of the Royal Society B: Biological Sciences 2022; 289:20221747
73. Jenkins F, Le T, Farhat R, et al. Validation of an HIV whole genome sequencing method for HIV drug resistance testing in an Australian clinical microbiology laboratory. Journal of Medical Virology 2023; 95:e29273
74. Bbosa N, Ssemwanga D, Ssekagiri A, et al. Phylogenetic and Demographic Characterization of Directed HIV-1 Transmission Using Deep Sequences from High-Risk and General Population Cohorts/Groups in Uganda. Viruses 2020; 12:331
75. Fogel JM, Bonsall D, Cummings V, et al. Performance of a high-throughput next-generation sequencing method for analysis of HIV drug resistance and viral load. Journal of Antimicrobial Chemotherapy 2020; 75:3510–3516
76. Hall M, Golubchik T, Bonsall D, et al. Demographics of sources of HIV-1 transmission in Zambia: a molecular epidemiology analysis in the HPTN 071 PopART study. The Lancet Microbe 2024; 5:e62–e71
77. Lin G-L, Golubchik T, Drysdale S, et al. Simultaneous Viral Whole-Genome Sequencing and Differential Expression Profiling in Respiratory Syncytial Virus Infection of Infants. The Journal of Infectious Diseases 2020; 222:S666–S671
78. Christensen KT, Pierard F, Bonsall D, et al. Phylogenetic Analysis of Hepatitis C Virus Infections in a Large Belgian Cohort Using Next-Generation Sequencing of Full-Length Genomes. Viruses 2023; 15:2391

79. Zhao L, Wymant C, Blanquart F, et al. Phylogenetic estimation of the viral fitness landscape of HIV-1 set-point viral load. Virus Evolution 2022; 8:veac022
80. Nascimento FF, Mehta SR, Little SJ, et al. Assessing transmission attribution risk from simulated sequencing data in HIV molecular epidemiology. AIDS 2024; 38:865
81. Capoferri AA, Lamers SL, Grabowski MK, et al. Recombination Analysis of Near Full-Length HIV-1 Sequences and the Identification of a Potential New Circulating Recombinant Form from Rakai, Uganda. AIDS Research and Human Retroviruses 2020; 36:467–474
82. Cuypers L, Thijssen M, Shakibzadeh A, et al. Signature of natural resistance in NS3 protease revealed by deep sequencing of HCV strains circulating in Iran. Infection, Genetics and Evolution 2019; 75:103966

Supplementary references

1. Ode H, Matsuda M, Matsuoka K, et al. Quasispecies Analyses of the HIV-1 Near-full-length Genome With Illumina MiSeq. Front Microbiol 2015; 6:1258
2. Gall A, Ferns B, Morris C, et al. Universal Amplification, Next-Generation Sequencing, and Assembly of HIV-1 Genomes. J Clin Microbiol 2012; 50:3838–3844
